# Characterization and Identification of Bioactive Polyphenols in the *Trapa* *bispinosa* Roxb. Pericarp Extract

**DOI:** 10.3390/molecules26195802

**Published:** 2021-09-24

**Authors:** Yuji Iwaoka, Shoichi Suzuki, Nana Kato, Chisa Hayakawa, Satoko Kawabe, Natsuki Ganeko, Tomohiro Uemura, Hideyuki Ito

**Affiliations:** 1 Department of Nutritional Science, Faculty of Health and Welfare Sciences, Okayama Prefectural University, Okayama 719-1197, Japan; iwaoka@fhw.oka-pu.ac.jp (Y.I.); ssho5532@gmail.com (S.S.); nana-k@m.ndsu.ac.jp (N.K.); chisa5835740fastriv@gmail.com (C.H.); satoko@mw.kawasaki-m.ac.jp (S.K.); ganeko@fukuyama-u.ac.jp (N.G.); 2 Hayashikane Sangyo Co., Ltd., 2-4-8 Yamatomachi, Shimonoseki 750-8608, Japan; tmuemura@hayashikane.co.jp

**Keywords:** *Trapa bispinosa* Roxb., polyphenol, ellagitannin, gallotannin, α-glucosidase inhibitor, advanced glycation end products (AGEs), antiglycation effect, LC/UV/ESIMS analysis

## Abstract

In this study, we present the isolation and characterization of the structure of six gallotannins (**1**–**6**), three ellagitannins (**7**–**9**), a neolignan glucoside (**10**), and three related polyphenolic compounds (gallic acid, **11** and **12**) from *Trapa bispinosa* Roxb. pericarp extract (TBE). Among the isolates, the structure of compound **10** possessing a previously unclear absolute configuration was unambiguously determined through nuclear magnetic resonance and circular dichroism analyses. The α-glucosidase activity and glycation inhibitory effects of the isolates were evaluated. Decarboxylated rugosin A (**8**) showed an α-glucosidase inhibitory activity, while hydrolyzable tannins revealed stronger antiglycation activity than that of the positive control. Furthermore, the identification and quantification of the TBE polyphenols were investigated by high-performance liquid chromatography coupled to ultraviolet detection and electrospray ionization mass spectrometry analysis, indicating the predominance of gallic acid, ellagic acid, and galloyl glucoses showing marked antiglycation properties. These findings suggest that there is a potential food industry application of polyphenols in TBE as a functional food with antidiabetic and antiglycation activities.

## 1. Introduction

Water chestnut (*Trapa bispinosa* Roxb.)—belonging to the family Lythraceae—is a floating annual aquatic plant originally distributed in Southeast Asia, and its cultivation is widely extended to Southern Europe, Africa, and Asia. The dried pericarp has been popular as a tea in the Fukuoka and Saga prefectures in Japan [1], and it has been used as a traditional folk medicine in China, such as an antidiarrheal and antipyretic agent [2]. The fruit and leaf extracts reportedly exhibit diverse biological, such as antioxidant [3] and anticancer [4] activities. The removal effect of industrial pollutant by this plant was reported as a sorbent [5]. The phytochemical studies on this plant revealed the presence of tannins, flavonoids, and saponins, while its detailed components remain elusive. The phytochemical and biological studies of *Trapa japonica* Flerov.—a species closely related to *Trapa bispinosa* Roxb.—described the isolation of ellagitannins (including trapanin, tellimagrandin II, trapanins A and B, rugosin D, and cornusiin G) and gallotannins (including 1,2,3- and 1,2,6-tri-*O*-galloyl-β-d-glucoses) from the leaves and pericarps, and the biological properties of the polyphenols [6,7,8,9]. Based on these studies, *Trapa bispinosa* Roxb. polyphenols are believed to contribute to various biological effects.

The accumulation of advanced glycation end products (AGEs) in the various tissues of our body is a cause of Alzheimer’s disease [10] and diabetes [11], suggesting that the inhibition of glycation reaction delays the chronic glycative stress-associated diseases. From a disease prevention perspective, food components and pharmaceutical ingredients that inhibit the α-glucosidase activity and glycation reaction are beneficial for our health. The *Trapa bispinosa* Roxb. pericarp extract (TBE) reportedly inhibits the α-glucosidase and glycation reactions [1,12]. However, the TBE components that contribute to these inhibitory effects remain largely unknown. In this study, with the aim of developing functional food materials for TBE that is popular as tea in Japan and effectively utilizing the pericarp which is often wasted, we isolated and characterized TBE polyphenols and evaluated the α-glucosidase and AGE-formation inhibitory effects of the isolated compounds. In addition, the TBE polyphenols showing α-glucosidase and AGE-formation inhibitory effects were identified and quantified by high-performance liquid chromatography coupled to ultraviolet detection and electrospray ionization mass spectrometry (LC/UV/ESIMS) analysis. As the results, we believe that this study might be attract the interest of the food industry due to the development and evaluation of potential application of TBE ingredients.

## 2. Results and Discussion

### 2.1. Isolation and Structural Elucidation of TBE-Derived Polyphenols and Related Compounds

**Figure 1:**
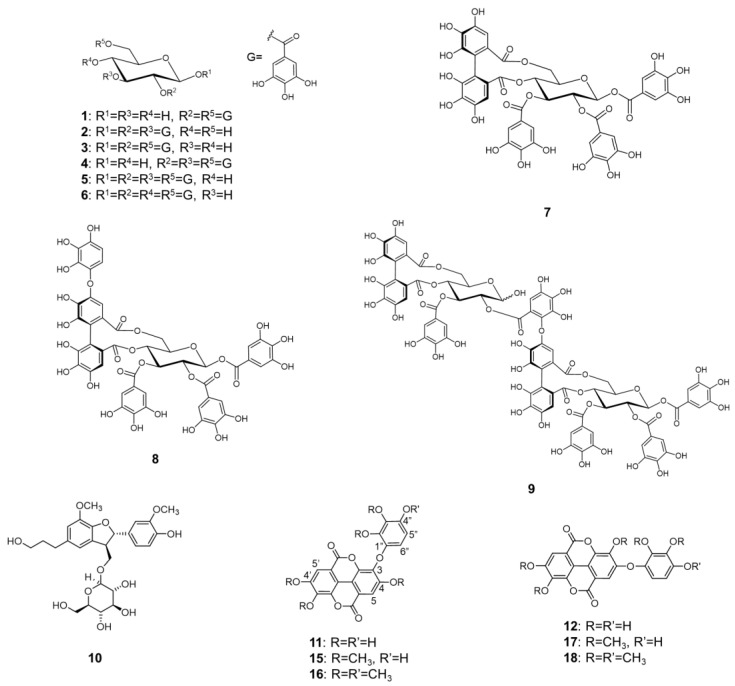
Chemical structures of the polyphenols isolated from TBE and the methylated compounds of **11** and **12**.

A 70% aqueous acetone extract of TBE was subsequently extracted with Et_2_O, EtOAc, and water-saturated *n*-BuOH. The EtOAc extract was repeatedly purified by Toyopearl HW-40 (coarse grade), MCI gel CHP20/P120, Sephadex LH-20, Mega Bond Elute C18 and preparative HPLC to obtain eight known compounds including gallic acid; six gallotannins as 2,6-di-*O*-galloyl-β-d-glucose (**1**) [13], 1,2,3-tri-*O*-galloyl-β-d-glucose (**2**) [14], 1,2,6-tri-*O*-galloyl-β-d-glucose (**3**) [14], 2,3,6-tri-*O*-galloyl-β-d-glucose (**4**) [15], 1,2,3,6-tetra-*O*-galloyl-β-d-glucose (**5**) [16], and 1,2,4,6-tetra-*O*-galloyl-β-d-glucose (**6**) [16]; and an ellagitannin as tellimagrandin II (**7**) [17]. The *n*-BuOH extract was subsequently subjected to column chromatography over Diaion HP-20 and Toyopearl HW-40 (coarse grade) to give compounds **5** and **7**. In a separate experiment, TBE aqueous solution was chromatographed over Diaion HP-20, Toyopearl HW-40 (coarse grade), MCI gel CHP20/P120, Bond Elute Plexa, and Mega Bond Elute C18 to give five known compounds including two ellagitannins as decarboxylated rugosin A (**8**) [18] and camptothin B (**9**) [7], a neolignan as (7′*S*,8′*R*)-dihydrodehydrodiconiferyl alcohol-9′-*O*-β-d-glucoside (**10**) [19], and two ellagic acid derivatives as rubuphenol (**11**) [20] and eschweilenol A (**12**) [21]. The known compounds were identified by direct comparison with authentic specimens or by comparison of spectroscopic data with those reported in the literature (Figure 1).

The structure of neolignan glucoside (**10**) was characterized as a known dihydrodehydrodiconiferyl alcohol-9′-*O*-glucoside [19] based on the 1D and 2D-nuclear magnetic resonance (NMR) analyses including ^1^H-^1^H correlation spectroscopy (COSY), heteronuclear single quantum correlation (HSQC), heteronuclear multiple bond correlation (HMBC), and nuclear Overhauser effect spectroscopy (NOESY) experiments (Figure S1) and electrospray ionization-mass spectrometry (ESI-MS) analysis. However, the absolute configuration in **10** was still unknown. The stereochemistry of glucose in **10** was confirmed to D-series since the released glucose by acid hydrolysis of **10** was a positive response to the reaction with glucose oxidase [22]. The coupling constant (*J*_7__′,8__′_ = 6.6 Hz) between the H-7′ and H-8′ protons suggested that the relative configuration of **10** at C-7′ and C-8′ was *threo* [23,24]. This relative configuration was further supported by the NOE correlations between the H-7′ and H-9′ protons, as well as those between the H-8′ and H-2′ and H-6′ protons. The absolute configuration of neolignan with dihydrobenzo[*b*]furan skeleton has been determined in agreement with the aromatic quadrant and *P*/*M* helicity rules [25,26].

**Figure 2:**
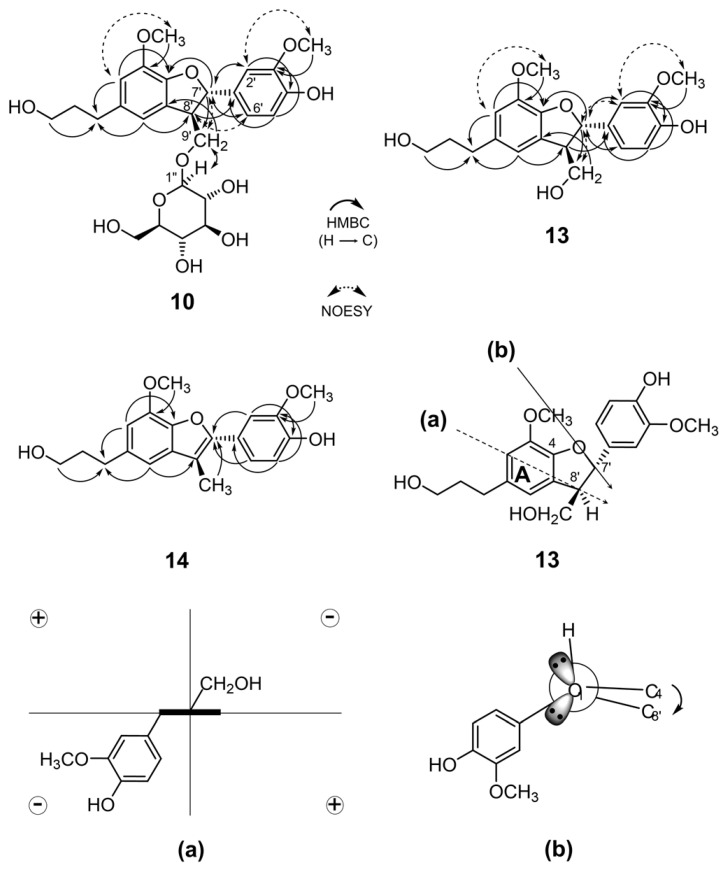
The key HMBC and NOESY correlations for compound **10**, compound **13** and **14** chemical structures, and projection for ^1^L_a_ (**a**) projection in the direction of the arrow, the wedge indicates the plane of the A-ring); and ^1^L_b_ (**b**) transition for **13**.

The structure of compound **13**, which was obtained by the hydrolysis of **10**, was confirmed based on ^1^H-NMR (Figure S2), ^13^C-NMR and atmospheric pressure ionization (APCI) MS analyses. The circular dichroism (CD) spectrum of **13** showed negative cotton at the ^1^L_a_ (around 230 nm), indicating that the absolute configuration of C-8′ was *R* series based on the aromatic quadrant rule. Furthermore, the *P*/*M* helicity rule provided evidence that the 7′*S*, 8′*R* configurations of **13** by showing positive cotton at ^1^L_b_ (around 290 nm) band in the CD spectrum. Based on these findings, the stereochemistry of **10** was determined as shown by the formula in Figure 2.

Compound **14** was also obtained as a side product from the hydrolysis of **10** (Figure 2 and Figure S2). The product has been reported to be an intermediate of adenosine A_1_ receptor ligand [27]. The production of **14** by acid hydrolysis of **10** was further supported the characterization of aglycone moiety of **10**.

Rubuphenol (**11**) and eschweilenol A (**12**) were identified by their ^1^H- and ^13^C-NMR, ^1^H-^1^H COSY, HSQC, HMBC, and NOESY experiments (Figure S3). The NOESY spectrum of **12** showed the correlation between H-5 and H-6″ protons, while that correlation was not observed in **11**. Compounds **11** and **12** were methylated to confirm the structures as the corresponding methylated derivatives (compounds **15**–**18**) based on spectral analyses (Figure S4). The ^1^H-^1^H COSY and NOESY spectra of compounds **15** and **16** showed the correlations between H-5 and 4-OCH_3_ protons, while those correlations were not observed in **17** and **18**. The observations of ^1^H-^1^H COSY and NOE correlations between H-5′ and 4′-OCH_3_ and, H-5″ and 4″-OCH_3_ protons in **16** and **18** supported the positions of hydroxyl group and ether linkage of **11** and **12**. Based on these findings, the structures of **11** and **12** were established as shown by the formulas in Figure 1. In this study, compounds **1**, **4**, **6**, **8**, **11**, and **12** were firstly isolated from *Trapa* species and the absolute configuration of **10** was confirmed based on NMR and CD analyses.

### 2.2. α-Glucosidase Inhibitory Activity of the TBE-Derived Compounds

The α-glucosidase inhibitory activities of the TBE-derived and the related polyphenols are shown in Table 1 as IC_50_ (μM) values. The inhibitory activities of gallic acid and ellagic acid, which are well-known metabolites of gallotannins and ellagitannins, respectively [28], showed little effect similar to that of gallotannins. (7′*S*,8′*R*)-Dihydrodehydrodiconiferyl alcohol-9′-*O*-β-d-glucoside (**10**) and its hydrolysates **13** and **14**, and rubuphenol (**11**) and eschweilenol A (**12**) also showed no inhibitory activity. Among the tested compounds, 1,2,3,4,6-penta-*O*-galloyl-β-d-glucose and decarboxylated rugosin A (**8**) showed inhibitory activity with IC_50_ values at 59.0 ± 0.4 μM and 20.7 ± 0.1 μM, respectively. However, the inhibitory activities of all tested compounds were not reached to that of acarbose as a positive inhibitor [29]. Tannin-containing plant extracts reportedly exert a strong α-glucosidase inhibitory effect [30], but little is known about the tannin contributors themselves. It has been reported that the extract of *Trapa japonica* belonging to the same genus as *T. bispinosa* and the isolated ellagitannin dimers cornusiin G and rugosin D from *T. japonica* showed the inhibitory activity on α-glucosidase comparable to that of acarbose [8]. The isolated polyphenols from *T. bispinosa* showed no effect, but the presence of cornusiin G was confirmed by HPLC analysis, which was described in Section 2.4, indicating cornusiin G and unidentified ellagitannin dimers might be contributed to inhibition on α-glucosidase. Further study is necessary to investigate the possibility that the other components besides ellagitannin dimers contribute to the activity.

### 2.3. Antiglycation Effects of the TBE-Derived Compounds

The inhibitory effect of the TBE-derived polyphenols and the related compounds on AGEs, generated by the glycation reaction between human serum albumin (HSA) and glucose or fructose was evaluated. All tested compounds exhibited significantly stronger AGE-formation inhibitory activity than the positive control aminoguanidine [31] with IC_50_ values in the range of 0.1 ± 0.0–14.7 ± 2.0 μM with glucose and of 0.2 ± 0.0–27.0 ± 2.6 μM with fructose (Table 2), except for (7′*S*, 8′*R*)-dihydrodehydrodiconiferyl alcohol-9′-*O*-β-d-glucoside (**10**) and its hydrolysates **13** and **14**. It is noteworthy that the gallotannin (**2**–**6**) and 1,2,3,4,6-penta-*O*-galloyl-β-d-glucose and ellagitannin (**7**–**9**) potencies were incomparably stronger than that of aminoguanidine.

AGEs are known to be generated via multiple pathways in glycation reactions [32]. Therefore, we also evaluated the AGE cross-link cleaving effects of the TBE polyphenols and the related compounds. Almost all tested compounds exhibited a stronger activity than the positive control *N*-phenacylthiazolium bromide (PTB) [33], except for ellagic acid, 2,6-di-*O*-galloyl-β-d-glucose (**1**), 1,2,3,4,6-penta-*O*-galloyl-β-d-glucose, **10**, **13**, and **14**. In particular, gallic acid, rubuphenol (**11**), and eschweilenol A (**12**) showed remarkable activities in this assay. Some of isolates have not been evaluated for antiglycation effects, since the isolated amount of the compounds were insufficient to test. However, these results indicated that the TBE-derived polyphenols exert AGE-formation inhibitory activity and might contribute to the antiglycative effect of TBE.

### 2.4. LC/UV/ESIMS Analysis of TBE

**Figure 3:**
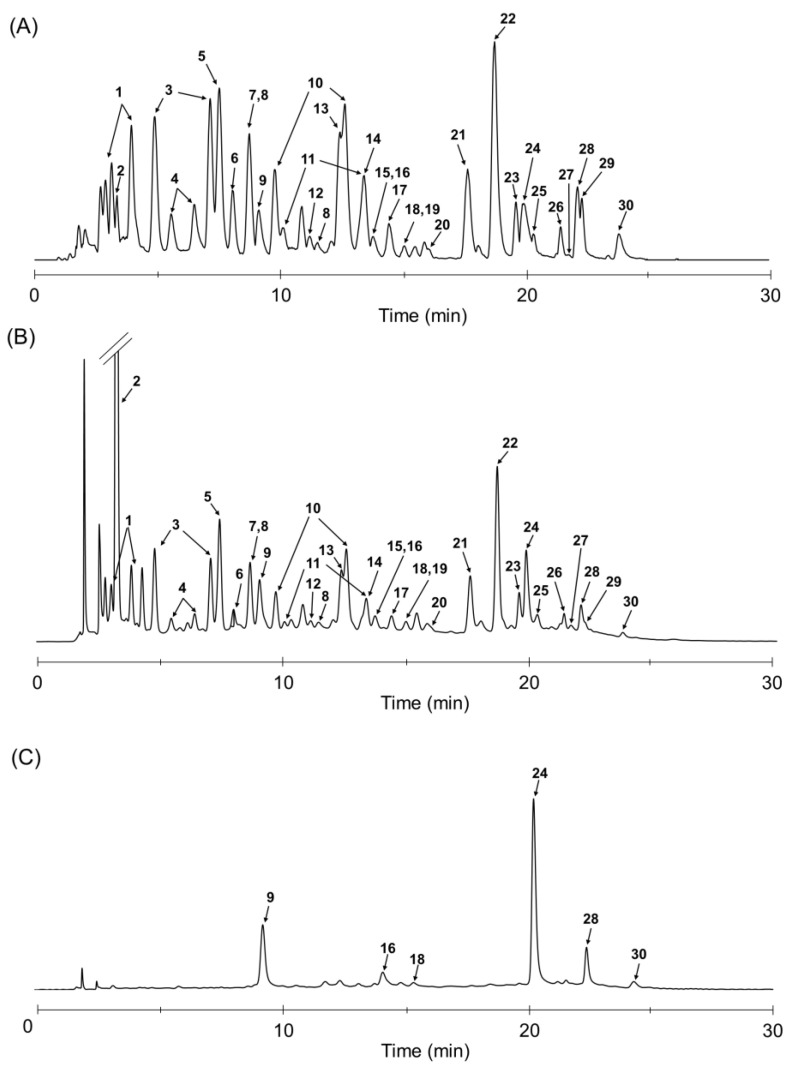
LC/UV/ESIMS analysis of TBE. Total ion chromatogram (**A**) and UV chromatogram at 280 nm (**B**) and 360 nm (**C**).

The presence of phenolic compounds, such as the gallotannins and ellagitannins, has already been previously reported in the pericarp of *Trapa* species [8,34]. However, the detailed polyphenol content in the pericarp of the *Trapa* species has not yet been revealed. There are few reports on the qualitative and quantification of hydrolyzable tannins containing both gallotannins and ellagitannins by LC-MS method. Here, we could identify and quantify a total of 30 polyphenols including gallotannins and ellagitannins in TBE by LC/UV/ESIMS method with each polyphenol specimen (Figure 3). The total ion and UV at 280 nm chromatograms of TBE displayed with good separation in Figure 3A,B, respectively. Among the candidates, compounds having lactones were clearly detected at UV at 360 nm (Figure 3C) for more separation and accurate quantification. In Table 3, gallic acid (32.2 ± 0.1 mg/g) exhibiting the most potent AGE cross-link cleaving activity among the isolated polyphenols is shown as a main TBE component, suggesting that it was produced from gallotannins or ellagitannins during TBE manufacturing, as well as ellagic acid (6.9 ± 0.1 mg/g). The various gallotannins possessing significant AGE-formation inhibitory activity were contained in the range of 0.2 ± 0.1–16.8 ± 1.2 mg/g. Valoneic acid dilactone (1.8 ± 0.2 mg/g), rubuphenol (**11**) (4.3 ± 0.1 mg/g), and eschweilenol A (**12**) (0.9 ± 0.2 mg/g) were minor TBE components, implying that these polyphenols were also ascribable to TBE ellagitannins. Urolithin M5 (1.4 ± 0.4 mg/g), a well-known ellagitannin metabolite, was also found in TBE. Urolithin M5 might be produced by biosynthesis in *Trapa bispinosa*, since urolithins A and B and isourolithin A reportedly contained in the plant of the same genus, *Trapa natans* [35]. Urolithin M5 has also been isolated from *Tamarix nilotica* [36]. The presence of ellagitannin dimers, camptothin B (**9**) and cornusiin G, and an ellagitannin monomer, 1,2-Di-*O*-galloyl-4,6-hexahydroxydiphenoyl-d-glucose in TBE could also be identified by LC/UV/ESIMS analysis. Gallotannins (**2**–**6**), tellimagrandin II (**7**), and decarboxylated rugosin A (**8**), which showed strong effects of both AGE-formation inhibition and AGE-derived crosslink cleaving, were contained in TBE at high levels. These results clearly provided the basic confirmation to the potential contribution of TBE polyphenols to antidiabetic and antiglycative effects.

## 3. Materials and Methods

### 3.1. Chemicals

The TBE was prepared as follows: the *Trapa bispinosa* pericarp cultivated in Thailand was dried, sterilized, and crushed at ambient conditions, followed by extraction with hot water (approximately six times the weight of the water chestnut pericarp). Dextrin was added to the extracted liquid so that the ratio of chestnut pericarp water extract to dextrin would be 67:33 using the dry weight. TBE was obtained after spray drying the extract and its moisture content was less than 10%. Ellagic acid, *N*-phenacylthiazolium bromide (PTB), and aminoguanidine hydrochloride were obtained from Wako Pure Chemical Industries (Osaka, Japan). 1-Phenyl-1,2-propanedione (PPD) and rat intestinal acetone powder were purchased from Sigma Aldrich (St Louis, MO, USA). Trimethylsilyldiazomethane (TMS-CHN_2_) was purchased from Tokyo Chemical Industry (Tokyo, Japan). Each polyphenol specimen was used compounds isolated from natural sources held in our library: gallotannins [37,38,39,40,41,42,43,44], 1,2-di-*O*-galloyl-4,6-hexahydroxydiphenoyl-d-glucose [45], cornusiin G [8], brevifolincarboxylic acid [39], urolithin M5 [46], and valoneic acid dilactone [41].

### 3.2. General Experimental Procedure

Optical rotations were recorded using a Jasco DIP-1000 polarimeter (Jasco, Tokyo, Japan). UV and CD spectra were measured by using Jasco V-530 spectrophotometer (Jasco, Tokyo, Japan) and Jasco J-710 spectropolarimeter (Jasco, Tokyo, Japan), respectively. ^1^H-NMR (600 MHz) and ^13^C-NMR (151 MHz) spectra including ^1^H-^1^H COSY, NOESY, HSQC, and HMBC were recorded on a Varian NMR system (Varian, Palo Alto, CA, USA) and chemical shifts are given in ppm (ppm) values relative to acetone-*d*_6_ (2.04 ppm for ^1^H and 29.8 ppm for ^13^C), CD_3_OD (3.35 ppm for ^1^H and 49.0 ppm for ^13^C), and CDCl_3_ (7.26 ppm for ^1^H and 77.0 ppm for ^13^C). ESI or APCI mass spectra were performed on a Bruker MicrOTOF II instrument (Bruker, Billerica, MA, USA) using direct sample injection. Reversed-phase HPLC was conducted on InertSustain C18 column (150 mm × 4.6 mm i.d., 5 μm, GL Sciences, Tokyo, Japan) at 40 °C with the mobile phase consisted of CH_3_CN:H_2_O:HCOOH (5:90:5) (solvent A) and CH_3_CN:H_2_O:HCOOH (45:50:5) (solvent B). The flow rate was 1.0 mL/min, and a linear gradient was programmed as follows: 0–15 min (solvent B: 10–30%), 15–20 min (solvent B: 30–50%), 20–30 min (solvent B: 10%) and the absorbance was monitored at 280 and 360 nm. Normal-phase HPLC was conducted on YMC-Pack SIL column (250 mm × 4.6 mm i.d., 5 μm, YMC, Kyoto, Japan) with *n*-hexane:MeOH:THF:HCOOH (55:33:11:1) containing oxalic acid (450 mg/L) by isocratic elution. The flow rate was 1.5 mL/min and the absorbance was monitored at 280 nm. Preparative HPLC was carried out under the same conditions as reversed-phase HPLC condition by using the mobile phase consisted of CH_3_CN:H_2_O:HCOOH (10:85:5) (condition 1) or MeOH:H_2_O:HCOOH (10:85:5) (condition 2). Column chromatography was carried out by Diaion HP-20 (Mitsubishi Chemical, Tokyo, Japan), Toyopearl HW-40 (coarse grade) (Tosoh, Tokyo, Japan), MCI gel CHP20/P120 (Mitsubishi Chemical, Tokyo, Japan), Sephadex LH-20 (GE Healthcare, Chicago, IL, USA), Mega Bond Elut C18 (Agilent technologies, Santa Clara, CA, USA), Bond Elut Plexa (Agilent technologies, Santa Clara, CA, USA), and YMC Gel ODS-AQ-HG (YMC, Kyoto, Japan).

### 3.3. Extraction and Isolation

TBE (450 g) was dissolved in H_2_O (700 mL) and the solution was extracted subsequently extracted with Et_2_O (3 × 700 mL), EtOAc (3 × 700 mL), and water-saturated *n*-BuOH (3 × 700 mL), to give Et_2_O (5.2 g), EtOAc (16.7 g), *n*-BuOH extracts (14.3 g), and H_2_O soluble portion (163.5 g). A part of EtOAc extract (5.0 g) was chromatographed over Toyopearl HW-40 (coarse grade) (40 cm × 2.2 i.d. cm) with 40%, 50%, 60%, and 70% aqueous MeOH—MeOH:H_2_O:acetone (7:2:1)—MeOH:H_2_O:acetone (7:1:2)—70% aqueous acetone in a stepwise elution mode. The 40% aqueous MeOH fraction gave gallic acid (779.9 mg), the 50% aqueous MeOH fraction gave 1,2,3-tri-*O*-galloyl-β-d-glucose (**2**) (148.1 mg), the 60% aqueous MeOH fraction gave 1,2,3,6-tetra-*O*-galloyl-β-d-glucose (**5**) (301.1 mg), and the 70% aqueous MeOH fraction gave 1,2,4,6-tetra-*O*-galloyl-β-d-glucose (**6**) (67.7 mg) and tellimagrandin II (**7**) (189.2 mg), by column chromatographic purification on each MCI gel CHP20/P120 (40 cm × 1.1 i.d. cm). The 40% aqueous MeOH fraction (200 mg) was purified by column chromatographies over MCI gel CHP20/P120 (40 cm × 1.1 i.d. cm) with aqueous MeOH, Sephadex LH-20 (40 cm × 1.1 i.d. cm) with EtOH-MeOH solvent system, Mega Bond Elut C18 cartridge column with aqueous MeOH, and preparative HPLC under the condition 1, to give 2,6-Di-*O*-galloyl-β-d-glucose (**1**) (3.0 mg). The 50% aqueous MeOH fraction (300 mg) was further purified by Mega Bond Elut C18 with 10%, 20%, 30%, 40%, 50%, 60%, and 70% aqueous MeOH–100% MeOH–70% aqueous acetone and preparative HPLC under the condition 2 to give 1,2,6-tri-*O*-galloyl-β-d-glucose (**3**) (0.8 mg) and 2,3,6-tri-*O*-galloyl-β-d-glucose (**4**) (0.9 mg). A part of *n*-BuOH extract (10 g) was chromatographed over Diaion HP-20 (40 cm × 5.0 i.d. cm) with H_2_O–10, 30, and 50% aqueous MeOH–100% MeOH–70% aqueous acetone in a stepwise elution mode. The 50% aqueous MeOH eluate (1.5 g) was further chromatographed over Toyopearl HW-40 (coarse grade) (40 cm × 2.2 i.d. cm) with 50%, 60%, and 70% aqueous MeOH–MeOH:H_2_O:acetone (7:2:1)–MeOH:H_2_O:acetone (7:1:2)–70% aqueous acetone in stepwise elution mode. Compounds **5** (40.3 mg) and **7** (43.8 mg) were obtained from the 60% aqueous MeOH and MeOH:H_2_O:acetone (7:2:1) fractions, respectively. TBE (500 g) was dissolved in H_2_O (10 L) and, the solution was subjected to Diaion HP-20 (80 cm × 5.0 i.d. cm) and eluted with H_2_O increasing amounts of MeOH (0–10–30–50–100% MeOH) and 70% aqueous acetone. A part (7.75 g) of the 50% aqueous MeOH eluate (23.0 g) was chromatographed over Toyopearl HW-40 (coarse grade) (40 cm × 2.2 i.d. cm) with 50, 60, and 70% aqueous MeOH–MeOH:H_2_O:acetone (7:2:1)–MeOH:H_2_O:acetone (7:1:2)–70% aqueous acetone as eluent. The combined fraction (400 mg) consisted of 70% aqueous MeOH and MeOH:H_2_O:acetone (7:1:2) fractions was purified by column chromatography over MCI gel CHP20/P120 (40 cm × 1.1 i.d. cm) with 30%, 40%, and 50% aqueous MeOH–100% MeOH–70% aqueous acetone. Subsequently, the 40% MeOH eluate (23.2 mg) was purified by Bond Elut Plexa with 30%, 40%, 50%, 60% aqueous MeOH and MeOH to give decarboxylated rugosin A (**8**) (8.4 mg) from the 60% aqueous MeOH eluate. The MeOH:H_2_O:acetone (7:2:1) eluate (120 mg) was purified by Mega Bond Elut C18 and YMC Gel ODS-AQ-HG (21 cm × 1.1 i.d. cm) with H_2_O-MeOH solvent system to give camptothin B (**9**) (7.3 mg). The 100% MeOH eluate (8.0 g) obtained by Diaion HP-20 separation was chromatographed over Toyopearl HW-40 (coarse grade) (43 cm × 2.2 i.d. cm) with H_2_O increasing amounts of MeOH (50–60–100% MeOH) followed by 70% aqueous acetone. (7′*S*, 8′*R*)-Dihydrodehydrodiconiferyl alcohol-9′-*O*-β-d-glucoside (**10**) (347.2 mg) was obtained from the 50% aqueous MeOH eluate. The fraction eluted with 100% MeOH and 70% aqueous acetone was dissolved in hot MeOH and the resulting solution was stored at 4 °C to remove ellagic acid as a precipitate. The collected supernatant (2.6 g) was chromatographed over Toyopearl HW-40 (coarse grade) (44 cm × 2.2 i.d. cm) with 70% aqueous MeOH–MeOH:H_2_O:acetone (7:2:1)–MeOH:H_2_O:acetone (7:1:2)–70% aqueous acetone. The MeOH:H_2_O:acetone (7:2:1)-MeOH:H_2_O:acetone (7:1:2) eluate was purified by MCI gel CHP20/P120 (24 cm × 1.1 i.d. cm) and YMC Gel ODS-AQ-HG (27 cm × 1.1 i.d. cm) with MeOH-H_2_O solvent system to afford rubuphenol (**11**) (33.1 mg) and eschweilenol A (**12**) (5.5 mg).

2,6-Di-*O*-galloyl-β-d-glucose (**1**): pale yellow amorphous powder; ^1^H-NMR [600 MHz, acetone-*d*_6_-D_2_O (9:1)] δ 7.11–7.14 (4H in total each, s, galloyl-H), 5.35 (1H, d, *J* = 3.6 Hz, Glc α-1), 4.88 (1H, t, *J* = 8.4 Hz, Glc β-2), 4.83 (1H, d, *J* = 7.8 Hz, Glc β-1), 4.74 (1H, dd, *J* = 3.6, 10.2 Hz, Glc α-2), 4.56 (1H, dd, *J* = 1.8, 11.4 Hz, Glc β-6), 4.51 (1H, dd, *J* = 1.8, 11.4 Hz, Glc α-6), 4.36 (2H, m, Glc α-6, β-6), 4.13 (1H, m, Glc α-5), 4.09 (1H, t, *J* = 9.6 Hz, Glc α-3), 3.74 (1H, t, *J* = 9 Hz, Glc β-3), 3.68 (1H, m, Glc β-5), 3.5–3.7 (Glc- and β-4, overlapped with DOH). HR-ESI-MS *m*/*z* 483.0787 [M − H]^−^ (calcd for C_20_H_20_O_14_-H, 483.0780).

1,2,3-Tri-*O*-galloyl-β-D-glucose (**2**): pale yellow amorphous powder; ^1^H-NMR [600 MHz, acetone-*d*_6_-D_2_O (9:1)] δ 7.09, 7.08, 7.00 (2H, each, s, galloyl-H), 6.06 (1H, d, *J* = 8.4 Hz, Glc H-1), 5.61 (1H, t, *J* = 9.6 Hz, Glc H-3), 5.40 (1H, dd, *J* = 8.4, 9.6 Hz, Glc H-2), 3.97 (1H, t, *J* = 9.6 Hz, Glc H-4), 3.93 (1H, d, *J* = 10.2 Hz, Glc H-6), 3.81 (2H, m, Glc H-5, 6). HR-ESI-MS *m*/*z* 635.0890 [M − H]^−^ (calcd for C_27_H_24_O_18_-H, 635.0890)

1,2,6-Tri-*O*-galloyl-β-d-glucose (**3**): pale yellow amorphous powder; ^1^H-NMR [600 MHz, acetone-*d*_6_-D_2_O (9:1)] δ 7.11, 7.07, 7.03 (2H, each, s, galloyl-H), 5.90 (1H, d, *J* = 9.0 Hz, Glc H-1), 5.22 (1H, t, *J* = 9.0 Hz, Glc H-2), 4.59 (1H, dd, *J* = 1.8, 12.0 Hz, Glc H-6), 4.37 (1H, dd, *J* = 5.4, 12.0 Hz, Glc H-6), 3.96 (1H, t, *J* = 9.0 Hz, Glc H-3), 3.91 (1H, m, Glc H-5), 3.71 (1H, t, *J* = 9.0 Hz, Glc H-4). HR-ESI-MS *m*/*z* 635.0898 [M − H]^−^ (calcd for C_27_H_24_O_18_-H, 635.0890).

2,3,6-Tri-*O*-galloyl-β-d-glucose (**4**): pale yellow amorphous powder; ^1^H-NMR [600 MHz, acetone-*d*_6_-D_2_O (9:1)] δ 7.00–7.14 (6H in total each, s, galloyl-H), 5.77 (1H, t, *J* = 9.6 Hz, Glc α-3), 5.47 (1H, d, *J* = 3.6 Hz, Glc α-1), 5.43 (1H, m, Glc β-3), 5.10 (1H, dd, *J* = 7.8, 9.6 Hz, Glc β-2), 5.01 (1H, d, *J* = 7.8 Hz, Glc β-1), 4.94 (1H, dd, *J* = 3.6, 9.6 Hz, Glc α-2), 4.59 (1H, d, *J* = 11.4 Hz, Glc β-6), 4.55 (1H, dd, *J* = 1.8, 12 Hz Glc α-6), 4.47–4.40 (3H, m, Glc α-6, β-6, β-5), 4.29 (1H, m, Glc α-5), 3.93 (1H, t, *J* = 9.6 Hz, Glc α-4), 3.88 (1H, d, *J* = 6 Hz, Glc β-4). HR-ESI-MS *m*/*z* 635.0892 [M − H]^−^ (calcd for C_27_H_24_O_18_-H, 635.0890).

1,2,3,6-Tetra-*O*-galloyl-β-d-glucose (**5**): pale yellow amorphous powder; ^1^H-NMR [600 MHz, acetone-*d*_6_-D_2_O (9:1)] δ 7.16, 7.09, 7.08, 7.01 (2H, each, s, galloyl-H), 6.14 (1H, d, *J* = 8.4 Hz, Glc H-1), 5.69 (1H, t, *J* = 9.6 Hz, Glc H-3), 5.49 (1H, dd, *J* = 8.4, 9.6 Hz, Glc H-2), 4.66 (1H, dd, *J* = 1.8, 12.6 Hz, Glc H-6), 4.50 (1H, dd, *J* = 5.4, 12.6 Hz, Glc H-6), 4.17 (1H, m, Glc H-5), 4.08 (1H, t, *J* = 9.6 Hz, Glc H-4). HR-ESI-MS *m*/*z* 787.1034 [M − H]^−^ (calcd for C_34_H_28_O_22_-H, 787.0999).

1,2,4,6-Tetra-*O*-galloyl-β-d-glucose (**6**): pale yellow amorphous powder; ^1^H-NMR [600 MHz, acetone-*d*_6_-D_2_O (9:1)] δ 7.14, 7.13, 7.09, 7.06 (2H, each, s, galloyl-H), 6.04 (1H, d, *J* = 8.4 Hz, Glc H-1), 5.40 (1H, t, *J* = 9.6 Hz, Glc H-4), 5.38 (1H, dd, *J* = 8.4, 9.6 Hz, Glc H-2), 4.53 (1H, dd, *J* = 1.2, 12.6 Hz, Glc H-6), 4.36 (1H, t, *J* = 9.6 Hz, Glc H-3), 4.30 (1H, m, Glc H-5), 4.20 (1H, dd, *J* = 5.4, 12.6 Hz, Glc H-6). HR-ESI-MS *m*/*z* 787.1008 [M − H]^−^ (calcd for C_34_H_28_O_22_-H, 787.0999).

Tellimagrandin II (**7**): pale yellow amorphous powder; ^1^H-NMR [600 MHz, acetone-*d*_6_-D_2_O (9:1)] δ 7.09, 6.99, 6.95 (2H, each, s, galloyl-H), 6.63, 6.47 [1H each, HHDP-H], 6.17 (1H, d, *J* = 7.8 Hz, Glc H-1), 5.81 (1H, t, *J* = 9.6 Hz, Glc H-3), 5.59 (1H, dd, *J* = 7.8, 9.6 Hz, Glc H-2), 5.32 (1H, dd, *J* = 6.6, 13.2 Hz, Glc H-6), 5.20 (1H, t, *J* = 9.6 Hz, Glc H-4), 4.53 (1H, dd, *J* = 6.6, 9.6 Hz, Glc H-5), 3.87 (1H, d, *J* = 13.2 Hz, Glc H-6). HR-ESI-MS *m*/*z* 937.0956 [M − H]^−^ (calcd for C_41_H_30_O_26_-H, 937.0953).

Decarboxylated rugosin A (**8**): dark brown amorphous powder; ^1^H-NMR [600 MHz, acetone-*d*_6_-D_2_O (9:1)] δ 7.08, 7.00, 6.98 (2H, each, s, galloyl-H), 6.49, 6.39 (1H each, s, decarboxylated valoneoyl group -H_a_, H_b_), 6.43, 6.38 (1H each, d, *J* = 9.0 Hz, decarboxylated valoneoyl group -H_c_, H_d_), 6.15 (1H, d, *J* = 7.8 Hz, Glc H-1), 5.81 (1H, t, *J* = 9.6 Hz, Glc H-3), 5.59 (1H, dd, *J* = 7.8, 9.6 Hz, Glc H-2), 5.27 (1H, dd, *J* = 6.6, 13.2 Hz, Glc H-6), 5.17 (1H, t, *J* = 9.6 Hz, Glc H-4), 4.13 (1H, dd, *J* = 6.6, 9.6 Hz, Glc H-5), 3.82 (1H, d, *J* = 13.2 Hz, Glc H-6). HR-ESI-MS *m*/*z* 1061.1114 [M − H]^−^ (calcd for C_47_H_34_O_29_-H, 1061.1113).

Camptothin B (**9**): dark brown amorphous powder; ^1^H-NMR [600 MHz, acetone-*d*_6_-D_2_O (9:1)] δ 7.09, 6.99, 6.97, 6.84 (4/3 H, each, s, galloyl-H), 7.08, 7.02, 6.98, 6.91 (2/3 H, each, s, galloyl-H), 7.11 (1/3H, s, valoneoyl-Hc), 7.00 (2/3H, s, valoneoyl-Hc), 6.65 (2/3H, s, HHDP-Hd), 6.64 (1/3H, s, HHDP-Hd), 6.498 (1/3H, s, HHDP-He), 6.496 (2/3H, s, HHDP-He), 6.62 (1/3H, s, valoneoyl-Ha), 6.59 (2/3H, s, valoneoyl-Ha), 6.18 (1/3H, s, valoneoyl-Hb), 6.16 (2/3H, s, valoneoyl-Hb), 6.18 (2/3H, d, *J* = 7.8 Hz, Glc H_R_-1), 6.16 (1/3H, d, *J* = 7.8 Hz, Glc H_R_-1), 5.80 (1/3H, t, *J* = 9.6 Hz, Glc H_L_-3), 5.60 (1/3H, t, *J* = 9.6 Hz, Glc H_R_-3), 5.59 (2/3H, t, *J* = 9.6 Hz, Glc H_R_-3), 5.55 (1/3H, dd, *J* = 7.8, 9.6 Hz, Glc H_R_-2), 5.54 (2/3H, dd, *J* = 7.8, 9.6 Hz, Glc H_R_-2), 5.46 (2/3H, t, *J* = 9.6 Hz, Glc H_L_-3), 5.44 (1/3H, dd, *J* = 6.6, 13.2 Hz, Glc H_L_-6), 5.35 (1/3H, dd, *J* = 4.2 Hz, Glc H_L_-1α), 5.27 (1/3H, dd, *J* = 6.6, 13.2 Hz, Glc H_R_-6), 5.21 (1/3H, d, *J* = 6.6, 13.2 Hz, Glc H_R_-6′), 5.17 (2/3H, dd, *J* = 6.6, 13.2 Hz, Glc H_L_-6), 5.12 (2/3H, dd, *J* = 8.4, 9.6 Hz, Glc H_L_-2), 5.09 (1/3H, t, *J* = 9.6 Hz, Glc H_L_-4), 5.07 (1/3H,2/3H t, *J* = 9.6 Hz, Glc H_R_-4), 5.06 (1/3H, t, *J* = 4.2, 9.6 Hz, Glc H_L_-2), 5.00 (2/3H, t, *J* = 9.6 Hz, Glc H_L_-4), 5.00 (2/3H, t, *J* = 9.6 Hz, Glc H_L_-4), 4.61 (1/3H, dd, *J* = 6.6, 9.6 Hz, Glc H_R_-5), 4.57 (1/3H, dd, *J* = 6.6, 9.6 Hz, Glc H_L_-5), 4.45 (2/3H, dd, *J* = 6.6, 9.6 Hz, Glc H_L_-4), 4.49 (2/3H, t, *J* = 8.4 Hz, Glc H_L_-1β), 3.93 (1/3H, d, *J* = 13.2 Hz, Glc H_L_-6), 3.87 (2/3H, d, *J* = 13.2 Hz, Glc H_R_-6′), 3.80 (2/3H, d, *J* = 13.2 Hz, Glc H_L_-6), 3.74 (1/3H, d, *J* = 13.2 Hz, Glc H_R_-6). HR-ESI-MS *m*/*z* 1721.1710 [M − H]^−^ (calcd for C_75_H_54_O_48_-H, 1721.1712).

(7′*S*,8′*R*)-Dihydrodehydrodiconiferyl alcohol-9′-*O*-β-d-glucoside (**10**): pale brown amorphous powder; ${\left[\alpha \right]}_{D}^{25}$ + 12.8° (*c* 0.5, MeOH); UV (MeOH) λ_max_ (log ε) 225 (4.30), 282 (3.92) nm; CD (MeOH) [α] (nm) − 4.3 × 10^3^ (223), +1.7 × 10^4^ (240), +1.0 × 10^4^ (291); ^1^H-NMR [600 MHz, CD_3_OD] δ 6.99 (1H, d, *J* = 1.8 Hz, H-2′), 6.84 (1H, dd, *J* = 1.8, 7.8 Hz, H-6′), 6.79 (1H, brs, H-6), 6.75 (1H, d, *J* = 7.8 Hz, H-5′), 6.71 (1H, brd, *J* = 1.2 Hz, H-2), 5.57 (1H, d, *J* = 6.6 Hz, H-7′), 4.35 (1H, d, *J* = 7.8 Hz, H-1″), 4.10 (1H, dd, *J* = 8.4, 9.6 Hz, H-9′a), 3.86 (1H, dd, *J* = 2.4, 12.0 Hz, H-6”a), 3.84 (3H, s, OCH_3_-3), 3.83 (1H, m, H-9′b), 3.81 (3H, s, OCH_3_-3′), 3.68 (1H, dd, *J* = 6.0, 12.0 Hz, H-6″a), 3.64 (1H, brdd, *J* = 6.6, 13.2 Hz, H-8′), 3.58 (2H, t, *J* = 6.6 Hz, H-9), 3.37 (1H, t, *J* = 9.0 Hz, H-3″), 3.31 (1H, t, *J* = 9.0 Hz, H-4″), 3.27 (1H, ddd, *J* = 2.4, 6.0, 9.0 Hz, H-5″), 3.23 (1H, dd, *J* = 7.8, 9.0 Hz, H-2″), 2.61 (2H, brt, *J* = 7.8 Hz, H-7), 1.80 (2H, m, H-8); ^13^C-NMR [151 MHz, CD_3_OD] δ 147.6 (C-3′), 146.00 (C-4′), 145.97 (C-4), 143.8 (C-3), 135.5 (C-1), 133.2 (C-1′), 128.3 (C-5), 118.4 (C-6′), 116.8 (C-6), 114.6 (C-5′), 112.6 (C-2), 109.4 (C-2′), 102.8 (C-1″), 87.8 (C-7′), 76.7 (C-3″), 76.6 (C-5″), 73.7 (C-2″), 70.9 (C-9′), 70.2 (C-4″), 61.3 (C-6″), 60.8 (C-9), 55.3 (C-3OCH_3_), 55.0 (C-3′OCH_3_), 51.5 (C-8′), 34.4 (C-8), 31.5 (C-7); HR-ESI-MS *m*/*z* 521.2041 [M − H]^−^ (calcd for C_26_H_34_O_11_-H, 521.2028).

Acid hydrolysis of **10**: A solution of **10** (50 mg) in 1 M HCl was heated in boiled water for 1 h. The reaction mixture was purified by Mega Bond Elut C18 with MeOH-H_2_O (0:100–10:90–20:80–30:70–40:60–50:50–60:40) in stepwise gradient. Compounds **13** (5.4 mg) and **14** (4.3 mg) were obtained from 40% and 60% MeOH fractions, respectively. The obtained H_2_O fraction was tested by Glucose CII Test Wako kit (Wako Pure Chemical Industries, Osaka, Japan) to determine D-series of glucose in **10** [22]. 

Compound **13** (aglycone of **10**): off-white amorphous powder; ${\left[\alpha \right]}_{D}^{25}$ + 14.1° (*c* 0.5, MeOH); UV (MeOH) λ_max_ (log ε) 227 (4.30), 286 (3.87) nm; CD (MeOH) [α] (nm) − 3.4 × 10^3^ (225), + 1.5 × 10^4^ (240), + 6.7 × 10^3^ (292); ^1^H-NMR [600 MHz, acetone-*d*_6_-D_2_O (9:1)] δ 6.99 (1H, d, *J* = 1.8 Hz, H-2′), 6.83 (1H, dd, *J* = 1.8, 7.8 Hz, H-6′), 6.78 (1H, d, *J* = 7.8 Hz, H-5′), 6.72 (1H, brs, H-6), 6.71 (1H, brs, H-2), 5.50 (1H, d, *J* = 6.6 Hz, H-7′), 3.82 (1H, m, H-9′a), 3.80 (3H, s, OCH_3_-3′), 3.78 (3H, s, OCH_3_-3), 3.73 (1H, dd, *J* = 7.2, 10.8 Hz, H-9′b), 3.52 (2H, t, *J* = 6.6 Hz, H-9), 3.47 (1H, brdd, *J* = 6.6, 13.2 Hz, H-8′), 2.58 (2H, brt, *J* = 7.8 Hz, H-7), 1.76 (2H, m, H-8); ^13^C-NMR [151 MHz, acetone-*d*_6_-D_2_O (9:1)] δ 147.6 (C-3′), 146.2 (C-4, 4′), 143.9 (C-3), 135.5 (C-1), 133.6 (C-1′), 128.9 (C-5), 118.4 (C-6′), 116.7 (C-6), 114.9 (C-5′), 112.8 (C-2), 109.7 (C-2′), 87.2 (C-7′), 63.6 (C-9′), 60.7 (C-9), 55.5 (C-3OCH_3_), 55.4 (C-3′OCH_3_), 54.0 (C-8′), 34.7 (C-8), 31.7 (C-7); HR-APCI-MS *m*/*z* 359.1499 [M − H]^−^ (calcd for C_20_H_24_O_6_-H, 359.1500).

Compound **14**: off-white amorphous powder; ^1^H-NMR [600 MHz, acetone-*d*_6_-D_2_O (9:1)] δ 7.32 (1H, d, *J* = 1.8 Hz, H-2′), 7.25 (1H, dd, *J* = 1.8, 8.4 Hz, H-6′), 6.97 (1H, d, *J* = 1.2 Hz, H-6), 6.95 (1H, d, *J* = 8.4 Hz, H-5′), 6.77 (1H, brd, *J* = 1.2 Hz, H-2), 3.97 (3H, s, OCH_3_-3), 3.91 (3H, s, OCH_3_-3′), 3.56 (2H, t, *J* = 6.6 Hz, H-9), 2.74 (2H, brt, *J* = 7.2 Hz, H-7), 2.39 (3H, s, H-9′), 1.86 (2H, m, H-8); ^13^C-NMR [151 MHz, acetone-*d*_6_-D_2_O (9:1)] δ 151.1 (C-7′), 147.8 (C-3′), 147.0 (C-4′), 144.7 (C-3), 141.0 (C-4), 137.9 (C-1), 132.9 (C-8′), 123.0 (C-5), 120.0 (C-6′), 115.4 (C-5′), 110.6 (C-6), 110.2 (C-2′), 109.5 (C-1′), 107.6 (C-2), 60.8 (C-9), 55.50 (C-3′OCH_3_), 55.46 (C-3OCH_3_), 34.9 (C-8), 32.3 (C-7), 8.8 (C-9′); HR-APCI-MS *m*/*z* 341.1394 [M − H]^−^ (calcd for C_20_H_2__2_O_5_-H, 341.1394).

Rubuphenol (**11**): off-white amorphous powder; ^1^H-NMR [600 MHz, CD_3_OD]: δ 7.53 (1H, s, H-5′), 7.33 (1H, s, H-5), 6.19 (1H, d, *J* = 9.0 Hz, H-6″), 6.16 (1H, d, *J* = 9.0 Hz, H-5′); ^13^C-NMR [151 MHz, CD_3_OD]: δ 160.7 (C-7), 160.6 (C-7′), 153.8 (C-4′), 149.5 (C-4), 143.4 (C-2 or 2′), 143.1 (C-4″), 141.2 (C-1″), 141.0 (C-3), 137.9 (C-3′), 137.15 (C-2”), 137.09 (C-2 or 2′), 135.5 (C-3″), 115.2 (C-6′), 113.4 (C-1), 113.3 (C-1′), 113.1 (C-5′), 111.8 (C-5), 108.9 (C-6), 107.4 (C-6″), 106.3 (C-5″); HR-ESI-MS *m*/*z* 425.0144 [M − H]^−^ (calcd for C_20_H_10_O_11_-H, 425.0150).

Eschweilenol A (**12**): off-white amorphous powder; ^1^H-NMR [600 MHz, CD_3_OD]: δ 7.50 (1H, s, H-5′), 7.30 (1H, s, H-5), 6.51 (1H, d, *J* = 9.0 Hz, H-6″), 6.40 (1H, d, *J* = 9.0 Hz, H-5″); ^13^C-NMR [151 MHz, CD_3_OD]: δ 161.34 (C-7′), 161.27 (C-7), 150.7 (C-4), 149.9 (C-4′), 145.0 (C-4″), 142.8 (C-3), 141.0 (C-3′), 139.7 (C-2″), 137.9 (2C, C-2, 2′) (2C), 137.4 (C-1″), 136.2 (C-3″), 115.9 (C-1), 113.6 (C-1′), 112.8 (C-6″), 111.9 (C-5), 111.8 (C-5′), 109.9 (C-6′), 108.7 (C-6), 107.3 (C-5″); HR-ESI-MS *m*/*z* 425.0134 [M − H]^−^ (calcd for C_20_H_10_O_11_-H, 425.0150).

Methylation of compounds **11** and **12**: Each solution of **11** (10 mg) or **12** (10 mg) in acetone was treated with an excess of TMS-CHN_2_ in hexane at room temperature overnight. Each reaction mixture was evaporated *in vacuo* and purified by preparative TLC (Merck, North Wales, PA, USA) with toluene:acetone (3:1) to obtain compounds **15** (Rf 0.55, 1.8 mg) and **16** (Rf 0.70, 2.1 mg) from **11**, and **17** (Rf 0.60, 0.7 mg) and **18** (Rf 0.74, 1.2 mg) from **12**, respectively.

Compound **15**: off-white amorphous powder; ^1^H-NMR [600 MHz, CDCl_3_]: δ 7.76 (1H, s, H-5′), 7.67 (1H, s, H-5), 6.52 (1H, d, *J* = 9.0 Hz, H-5″), 6.23 (1H, d, *J* = 9.0 Hz, H-6″), 4.24, 4.032, 4.026, 4.02, 3.95 (each 3H, s, OCH_3_-H); HR-APCI-MS *m*/*z* 497.1102 [M + H]^+^ (calcd for C_25_H_20_O_11_ + H, 497.1078).

Compound **16**: off-white amorphous powder; ^1^H-NMR [600 MHz, CDCl_3_]: δ 7.76 (1H, s, H-5′), 7.67 (1H, s, H-5), 6.47 (1H, d, *J* = 9.0 Hz, H-5″), 6.41 (1H, d, *J* = 9.0 Hz, H-6″), 4.24, 4.03, 4.00 (each 3H, s, OCH_3_-H), 3.94 (6H, s, OCH_3_-H), 3.82 (3H, s, OCH_3_-H); HR-APCI-MS *m*/*z* 511.1258 [M + H]^+^ (calcd for C_26_H_22_O_11_ + H, 511.1235).

Compound **17**: off-white amorphous powder; ^1^H-NMR [600 MHz, CDCl_3_] δ 7.71 (1H, s, H-5′), 7.49 (1H, s, H-5), 6.79 (1H, d, *J* = 9.0 Hz, H-6″), 6.75 (1H, d, *J* = 9.0 Hz, H-5″), 4.32, 4.21, 4.04, 3.98, 3.83 (each 3H, s, OCH_3_-H); HR-APCI-MS *m*/*z* 497.1065 [M + H]^+^ (calcd for C_25_H_20_O_11_ + H, 497.1078).

Compound **18**: off-white amorphous powder; ^1^H-NMR [600 MHz, CDCl_3_] δ 7.71 (1H, s, H-5′), 7.47 (1H, s, H-5), 6.85 (1H, d, *J* = 9.0 Hz, H-6″), 6.69 (1H, d, *J* = 9.0 Hz, H-5″), 4.32, 4.20, 4.04, 3.92, 3.91, 3.82 (each 3H, s, OCH_3_-H); HR-APCI-MS *m*/*z* 511.1245 [M + H]^+^ (calcd for C_26_H_22_O_11_ + H, 511.1235).

### 3.4. α-Glucosidase Inhibitory Activity

The α-glucosidase inhibitory activity was tested according to the previously described method by Kirino et al. [47] with a slight modification. Rat intestinal acetone powder was mixed with 0.1 M phosphate buffer (pH 7) and centrifuged at 18,500× *g* and 4 °C for 20 min. The resulting supernatant was collected and used as a glucosidase solution for enzymatic assay. Each sample solution (160 μL) was mixed with 250 mM maltose solution (20 μL) in 0.2 M phosphate buffer (pH 7) and then incubated at 37 °C for 3 min. After incubation, the enzymatic reaction was started by adding glucosidase solution from the rat intestine (20 μL) and the resulting reaction mixtures were further incubated at 37 °C for 15 min. After 15 min, the reaction mixtures were immediately heated at 100 °C for 5 min to stop the reaction followed by cooling on ice for 5 min. The amount of glucose in the reaction mixtures was determined using the F-Kit glucose (Roche diagnostics, Co., Tokyo, Japan) by measuring the absorbance at 340 nm. A control was carried out with 0.1 M phosphate buffer (pH 7) instead of sample solution. For the blank, the glucosidase solution was replaced with distilled water. The glucosidase inhibitory rates of tested samples were calculated as;

Inhibitory rate (%) = 100 − [(*A*_sample_ − *A*_blank_)/(*A*_control_ − *A*_blank_)] × 100 (1)

where *A*_sample_, *A*_control_, and *A*_blank_ is the absorbance of the tested sample, control, and blank, respectively. The experimental data are represented as IC_50_ (μM) values.

### 3.5. Inhibitory Effect on AGE-Formation 

The antiglycation effects of compounds isolated from TBE and several other related compounds were evaluated based on their AGE inhibitory activities as described previously [25], with slight modifications. Briefly, the sample solution was added to a reaction mixture containing 83.3 mM phosphate buffer (pH 7.2), 2.0 M glucose, 2.0 M fructose, 8.0 mg/mL human serum albumin (HSA), and distilled water (6:1:1:1:1, *v*/*v*). As a control, the vehicle was supplemented instead of the sample solution. For each blank, glucose or fructose was replaced with distilled water, and the total volume was set to 1000 μL. After incubation of the sample mixture at 60 °C for 40 h, the solutions were diluted 8-fold with distilled water, dispensed into a black microplate in 200 µL portions, and their fluorescence intensities were measured at excitation and emission wavelengths of 370 and 465 nm, respectively, using a Power Scan HT (DS Pharma Biomedical Co. Ltd., Osaka, Japan). The inhibitory rate was calculated as;

Inhibitory rate (%) = 100 − [(*S* − *SB*)/(*C* − *CB*)] × 100 (2)

where *S* is the relative intensity of the sample solution, *C* is the relative intensity of the control solution, and *SB* and *CB* are the intensities of the glucose or fructose-omitted blank solutions. The experimental data are represented as IC_50_ (μM) values.

### 3.6. AGE-Derived Crosslink-Cleaving Effect

The AGE crosslink-cleaving activity of the same samples was evaluated according to the previously described method by Kato et al. [28] with a slight modification. Briefly, the 1 mg/mL of tested samples prepared with H_2_O were mixed with 1.13 mM PPD solution in MeOH:50 mM phosphate buffer (pH 7.4) (1:1) and incubated at 37 °C for 4 h. After 4 h, the reaction was stopped with 200 μL of 2 M HCl, then the stopped reaction mixtures were centrifuged at 8200× *g* for 5 min. The amount of benzoic acid in the supernatant was measured by reversed-phase HPLC. The following conditions were applied: column, InertSustain C18 column (150 mm × 4.6 i.d mm., 5 μm); mobile phase, 50 mM phosphate buffer (pH 2.2):CH_3_CN (80:20) (solvent A) and 50 mM phosphate buffer (pH 2.2):CH_3_CN (50:50) (solvent B), the gradient program, 0–20 min (solvent B: 0–25%), 20–25 min (solvent B: 25–100%), 25–36 min (solvent B: 100–25%); column temperature, 40 °C; detection, UV at 230 nm; flow rate, 1.0 mL/min. These cleaving effects were calculated as equivalents to benzoic acid.

### 3.7. TBE Polyphenol Identification and Quantification by LC/UV/ESIMS Analysis

TBE (1.0 g) was sonicated with 70% aqueous acetone (3 × 10 mL) and the resulting suspension was centrifuged at 2200× *g* for 5 min. The supernatant was collected and dried. The obtained acetone extract from TBE (0.68 g) was dissolved in 50% aqueous MeOH to a concentration of 5 mg/mL and the solution of TBE extract was subjected to LC/UV/ESIMS. This analysis was performed on Waters 2695 separation module (Waters, Milford, MA, USA) coupled to Shimadzu SPD-6AV UV–vis spectrophotometric detector (Shimadzu, Kyoto, Japan) and Bruker MicrOTOF II instrument equipped with ESI source. The analyte was separated by InertSustain C18 column (150 mm × 4.6 mm i.d., 5 μm) at 40 °C with the mobile phase consisted of CH_3_CN:H_2_O:HCOOH (94.5:5:0.1) (solvent A) and CH_3_CN:H_2_O:HCOOH (54.9:45:0.1) (solvent B). The flow rate was 1.0 mL/min (splitting flow rate at 0.2 mL/min to MS unit), and a linear gradient was programmed as follows: 0–15 min (solvent B: 10–30%), 15–20 min (solvent B: 30–50%), 20–30 min (solvent B: 10%) and UV detection was monitored at 280 nm and 360 nm. MS parameters in negative ion mode were as follows: capillary voltage, 3.5 kV; nebulizer, 0.4 bar; dry gas, 4.0 L/min; dry temperature, 180 °C. The MS spectra were recorded in the range of *m*/*z* 50–3000. The polyphenol contents were expressed as mg/g (dry weight) by the absolute calibration curve method based on UV chromatogram.

## 4. Conclusions

In summary, we isolated the 13 known polyphenolic compounds including gallic acid, six galloyl glucoses (**1**–**6**), three ellagitannins (**7**–**9**), one neolignan (**10**), and ellagic acid derivatives **11** and **12** from TBE. The absolute configuration of **10** was confirmed by the aromatic quadrant and *P*/*M* helicity rules based on our CD analysis. Among the isolated polyphenols, decarboxylated rugosin A (**8**) and 1,2,3,4,6-penta-*O*-galloyl-β-d-glucose showed α-glucosidase inhibitory activities. Gallotannins and ellagitannins showed more significant inhibitory effect on AGE formation than that of gallic acid. Furthermore, gallic acid showed most potent AGE-derived crosslink cleaving activity among the tested polyphenols. A total of 30 TBE polyphenols were comprehensively identified by LC/UV/ESIMS analysis. The contents of tannins and the related polyphenols were also analyzed using LC/UV/ESIMS, indicating that gallic acid and gallotannins showing antiglycation effects were contained the major level in TBE. Further investigations are required to develop a deeper understanding of the TBE antidiabetic and antiglycation effects as well as the safety by in vivo experiments or clinical trial. Since the pericarp of this plant has experience in food as tea, it is considered that safety is guaranteed to some extent. The results of this study suggested the TBE polyphenols are a good source for antidiabetic and antiglycation effects, which could be applied as functional foods or nutritional supplements to improve human health benefits.

## Figures and Tables

**Table 1:** α-Glucosidase inhibitory activity of polyphenols isolated from TBE and related compounds.

Compound	Inhibitory Effects on α-Glucosidase Activity
	IC_50_ (μM)
Gallic acid [8]	>100
Ellagic acid	>100
1,2,3-Tri-*O*-galloyl-β-d-glucose (**2**) [8]	>100
1,2,6-Tri-*O*-galloyl-β-d-glucose (**3**) [8]	>100
1,2,3,6-Tetra-*O*-galloyl-β-d-glucose (**5**) [8]	>100
1,2,4,6-Tetra-*O*-galloyl-β-d-glucose (**6**)	>100
1,2,3,4,6-Penta-*O*-galloyl-β-d-glucose	59.0 ± 0.4
Tellimagrandin II (**7**) [8]	>100
Decarboxylated rugosin A (**8**)	20.7 ± 0.1
Camptothin B (**9**)	>100
Compound **10**	>100
Compound **13**	>100
Compound **14**	>100
Rubuphenol (**11**)	>100
Eschweilenol A (**12**)	>100
Cornusiin G [8]	6.3 ± 0.1
Acarbose	4.0 ± 0.1

Data are expressed as the means ± SE (*n* = 3).

**Table 2:** AGE-formation inhibitory effects in HSA/glucose or fructose and AGE-derived crosslink-cleaving activities of polyphenols isolated from TBE and related compounds.

Compound	Inhibitory Effects on AGE Formation		Crosslink-Cleaving Activities
	IC_50_ (μM)		
	Glucose	Fructose	Relative Ratio *
Gallic acid	14.7 ± 2.0	27.0 ± 2.6	720.1 ± 54.1
Ellagic acid	1.8 ± 0.1	2.5 ± 0.1	11.4 ± 0.1
2,6-Di-*O*-galloyl-β-d-glucose (**1**)	1.5 ± 0.1	3.3 ± 0.0	77.4 ± 4.0
1,2,3-Tri-*O*-galloyl-β-d-glucose (**2**)	0.4 ± 0.0	0.4 ± 0.0	190.5 ± 2.5
1,2,6-Tri-*O*-galloyl-*β*-d-glucose (**3**)	0.3 ± 0.0	0.4 ± 0.0	146.6 ± 12.6
2,3,6-Tri-*O*-galloyl-*β*-d-glucose (**4**)	0.3 ± 0.0	1.0 ± 0.0	N.T.
1,2,3,6-Tetra-*O*-galloyl-β-d-glucose (**5**)	0.3 ± 0.0	0.3 ± 0.0	209.0 ± 33.7
1,2,4,6-Tetra-*O*-galloyl-β-d-glucose (**6**)	0.3 ± 0.0	0.3 ± 0.0	159.6 ± 12.7
1,2,3,4,6-Penta-*O*-galloyl-β-d-glucose	0.2 ± 0.0	0.2 ± 0.0	97.9 ± 2.1
Tellimagrandin II (**7**)	0.2 ± 0.0	0.3 ± 0.0	230.8 ± 12.2
Decarboxylated rugosin A (**8**)	0.3 ± 0.0	0.3 ± 0.0	233.0 ± 5.8
Camptothin B (**9**)	0.1 ± 0.0	0.2 ± 0.0	180.6 ± 4.2
Compound **10**	>500	>1000	16.7 ± 1.4
Compound **13**	>500	>1000	17.2 ± 1.1
Compound **14**	>500	>1000	0.5 ± 1.1
Rubuphenol (**11**)	2.4 ± 0.0	4.7 ± 0.3	514.8 ± 11.2
Eschweilenol A (**12**)	2.4 ± 0.0	2.9 ± 0.1	484.5 ± 12.5
Aminoguanidine	258.9 ± 6.8	801.0 ± 17.7	N.T.
*N*-Phenacylthiazolium bromide (PTB)	N.T.	N.T.	100

Data are expressed as the means ± SE (*n* = 3), N.T. means not tested, * The concentrations of the tested samples are 100 μg/mL.

**Table 3:** TBE polyphenol content.

Peak No.	Compound	t_R_ (min)	MS (*m*/*z*)	Content (mg/g of Dry Weight)
1	2,3-Di-*O*-galloyl-β-d-glucose	3.18, 3.94	483 [M − H]^−^	15.5 ± 0.2
2	Gallic acid	3.23	339 [2M − H]^−^	32.2 ± 0.1
3	2,6-Di-*O*-galloyl-β-d-glucose (**1**)	4.90, 7.52	483 [M − H]^−^	N.T.
4	3,6-Di-*O*-galloyl-β-d-glucose	5.55, 6.58	483 [M − H]^−^	3.9 ± 0.0
5	1,6-Di-*O*-galloyl-β-d-glucose	7.53	483 [M − H]^−^	16.8 ± 1.2
6	Digalloyl glucose	8.07	483 [M − H]^−^	N.T.
7	1,2,3-Tri-*O*-galloyl-β-d-glucose (**2**)	8.75	635 [M − H]^−^	4.8 ± 0.0
8	3,4,6-Tri-*O*-galloyl-β-d-glucose	8.75, 11.6	635 [M − H]^−^	3.4 ± 0.1
9	Brevifolincarboxylic acid	9.22	291 [M − H]^−^	1.7 ± 0.1
10	2,3,6-Tri-*O*-galloyl-β-d-glucose (**4**)	9.86, 12.6	635 [M − H]^−^	N.T.
11	2,4,6-Tri-*O*-galloyl-β-d-glucose	10.2, 13.5	635 [M − H]^−^	0.2 ± 0.1
12	Trigalloyl glucose	11.3	635 [M − H]^−^	N.T.
13	1,2,6-Tri-*O*-galloyl-β-d-glucose (**3**)	12.5	635 [M − H]^−^	4.1 ± 0.5
14	1,3,6-Tri-*O*-galloyl-β-d-glucose	13.5	635 [M − H]^−^	1.8 ± 0.7
15	1,2-Di-*O*-galloyl-4,6-hexahydroxydiphenoyl-β-d-glucose	13.8	785 [M − H]^−^	0.9 ± 0.0
16	Valoneic acid dilactone	13.9	469 [M − H]^−^	1.8 ± 0.2
17	Trigalloyl glucose	14.5	635 [M − H]^−^	N.T.
18	Urolithin M5	15.1	275 [M − H]^−^	1.4 ± 0.4
19	1,4,6-Tri-*O*-galloyl-β-d-glucose	15.1	635 [M − H]^−^	0.6 ± 0.0
20	Camptothin B (**9**)	16.2	860 [M − 2H]^2−^	N.T.
21	Tellimagrandin II (**7**)	17.7	937 [M − H]^−^	5.7 ± 0.0
22	1,2,3,6-Tetra-*O*-galloyl-β-d-glucose (**5**)	18.8	787 [M − H]^−^	13.3 ± 0.0
23	1,2,4,6-Tetra-*O*-galloyl-β-d-glucose (**6**)	19.7	787 [M − H]^−^	1.5 ± 0.0
24	Ellagic acid	19.9	301 [M − H]^−^	6.9 ± 0.1
25	Decarboxylated rugosin A (**8**)	20.3	1061 [M − H]^−^	2.4 ± 0.0
26	1,2,3,4,6-Penta-*O*-galloyl-β-d-glucose	21.5	939 [M − H]^−^	0.7 ± 0.0
27	Cornusiin G	21.7	861 [M − 2H]^2−^	0.3 ± 0.0
28	Rubuphenol (**11**)	22.2	425 [M − H]^−^	4.3 ± 0.1
29	Compound **10**	22.1	521 [M − H]^−^	1.3 ± 0.1
30	Eschweilenol A (**12**)	24.0	425 [M − H]^−^	0.9 ± 0.2

Data are expressed as the means ± SE (*n* = 3), N.T. means not tested, t_R_: retention time.

## Data Availability

Data is contained within the article and supplementary materials.

## Supplementary Materials

The following are available online, Figure S1: 1D and 2D-NMR spectra of (7′*S*, 8′*R*)-dihydrodehydrodiconiferyl alcohol-9′-*O*-β-d-glucoside (**10**); Figure S2: ^1^H-NMR spectrum of compounds **13** and **14**; Figure S3: 1D and 2D-NMR spectra of rubuphenol (**11**) and eschweilenol A (**12**); Figure S4: 1D and 2D-NMR spectra of compounds **15**–**18**.

## Sample Availability

Not available.
