# Supplementary material for: Characterization and Identification of Bioactive Polyphenols in the Trapa bispinosa Roxb. Pericarp Extract
Source: Molecules. 2021 Sep 24;26(19):5802. doi: 10.3390/molecules26195802 (PMC8510478; doi:10.3390/molecules26195802)
Supplement: Supplementary file 1 [file molecules-26-05802-s001.zip › molecules-1360153-supplementary.pdf]

Characterization and identification of bioactive polyphenols in the *Trapa bispinosa* Roxb. pericarp extract

Yuji Iwaoka[1], Shoichi Suzuki[1], Nana Kato[1], Chisa Hayakawa[1], Satoko Kawabe[1], Natsuki Ganeko[1], Tomohiro Uemura[2], and Hideyuki Ito[1,*]

[1]*Department of Nutritional Science, Faculty of Health and Welfare Sciences, Okayama Prefectural University, 111 Kuboki, Soja, Okayama 719-1197, Japan*

[2]*Hayashikane Sangyo Co., Ltd. 2-4-8 Yamatomachi, Shimonoseki, Yamaguchi 750-8608, Japan*

*Corresponding author:

Hideyuki Ito

Department of Nutritional Science, Faculty of Health and Welfare Sciences, Okayama Prefectural University, 111 Kuboki, Soja, Okayama 719-1197, Japan.

e-mail: hito@fhw.oka-pu.ac.jp

Supporting Information

Figure. S1. 1D and 2D-NMR spectra of (7'*S*, 8'*R*)-dihydrodehydrodiconiferyl alcohol-9'-*O*-β-D-glucoside (**10**). ^{1}H-NMR (A), ^{13}C-NMR (B), HSQC (C), HMBC (D), and NOESY (E) spectra.

Figure. S2. ^{1}H-NMR spectrum of compounds **13** (A) and **14** (B).

Figure. S3. 1D and 2D-NMR spectra of rubuphenol (**11**) and eschweilenol A (**12**). ^{1}H-NMR (A), and NOESY (B) spectra.

Figure. S4. 1D and 2D-NMR spectra of compounds **15**–**18**. ^{1}H-NMR (A), ^{1}H-^{1}H COSY (B), and NOESY (C) spectra.

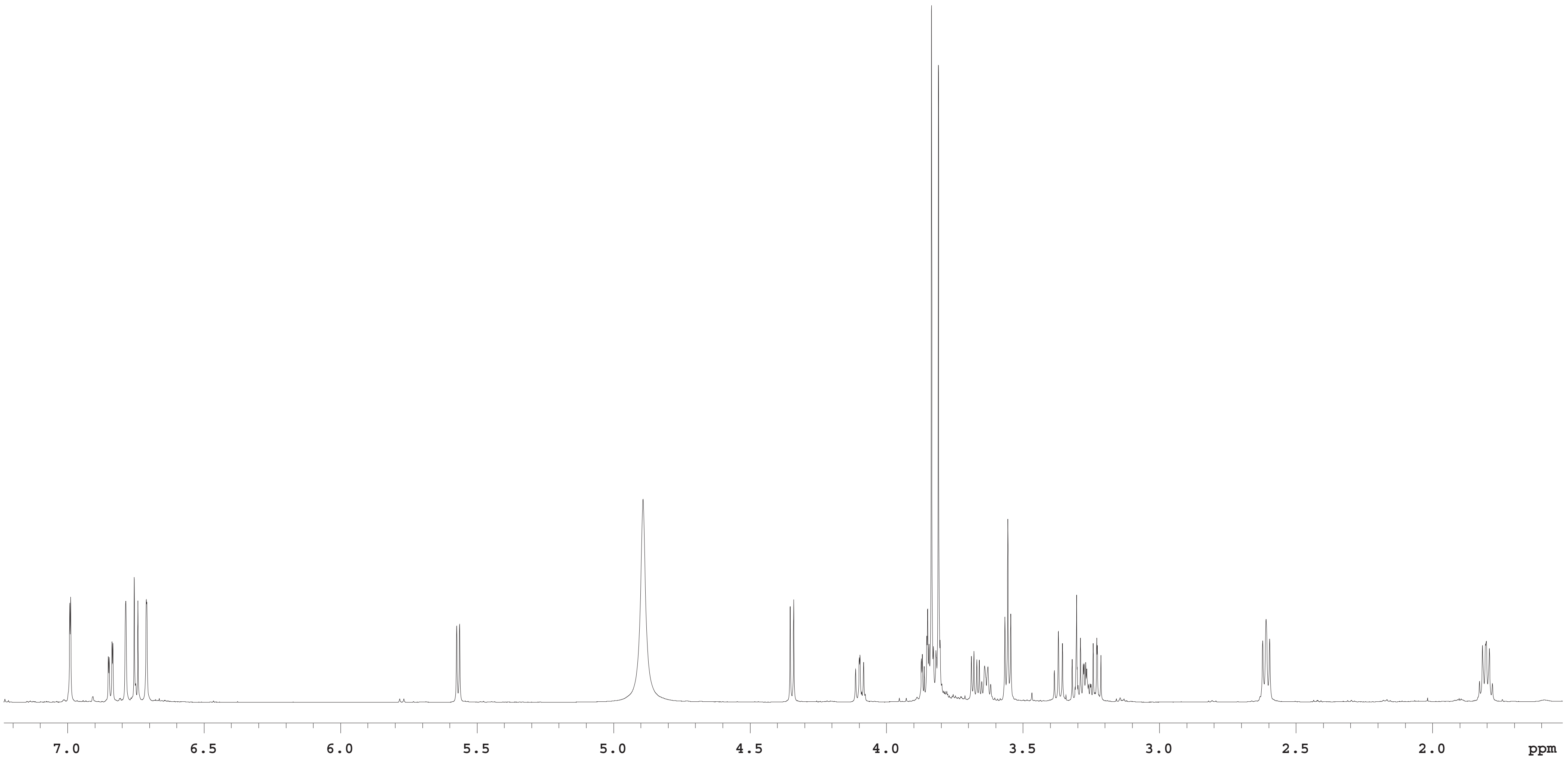

Figure.S1A

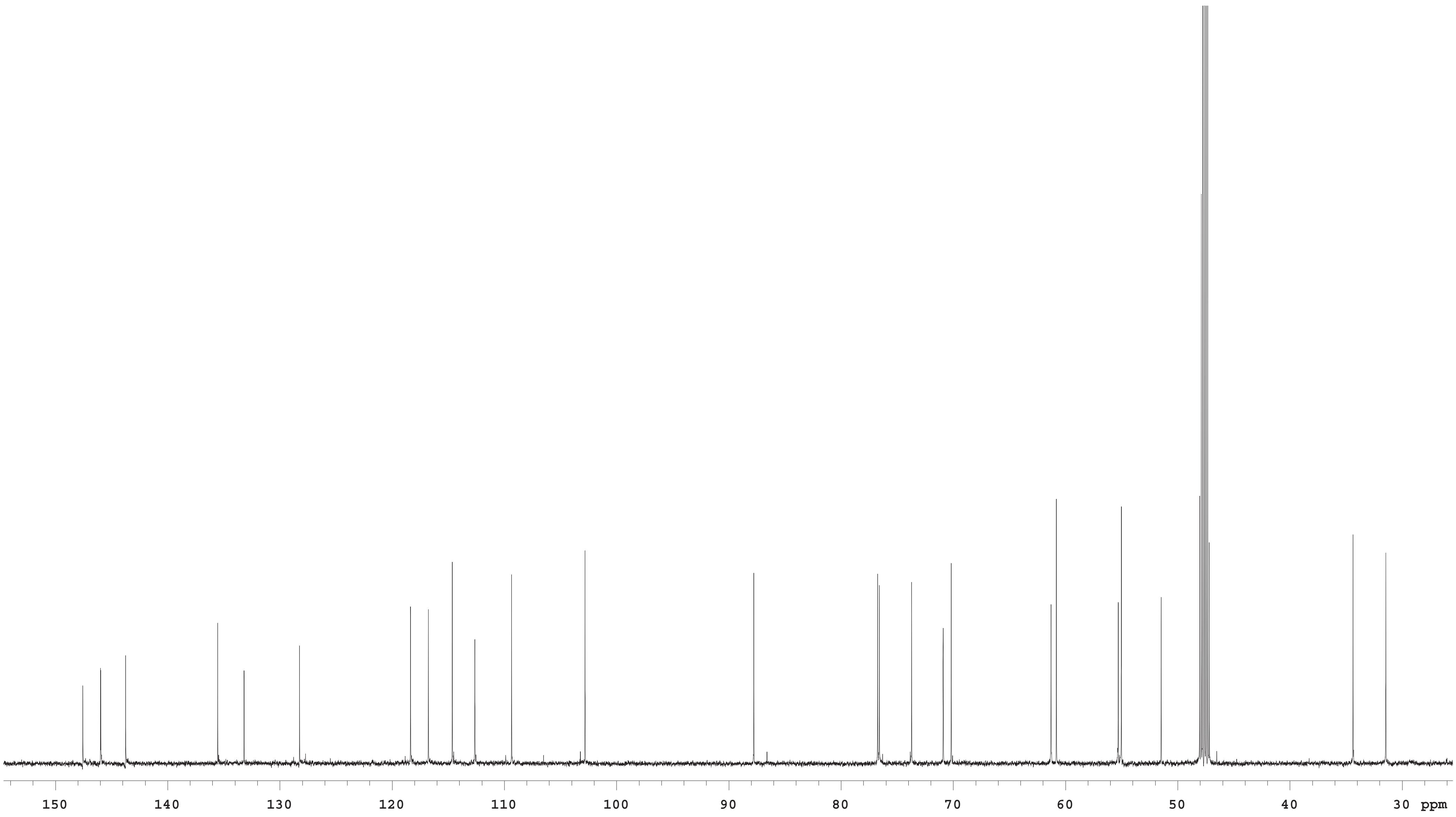

Figure. S1B

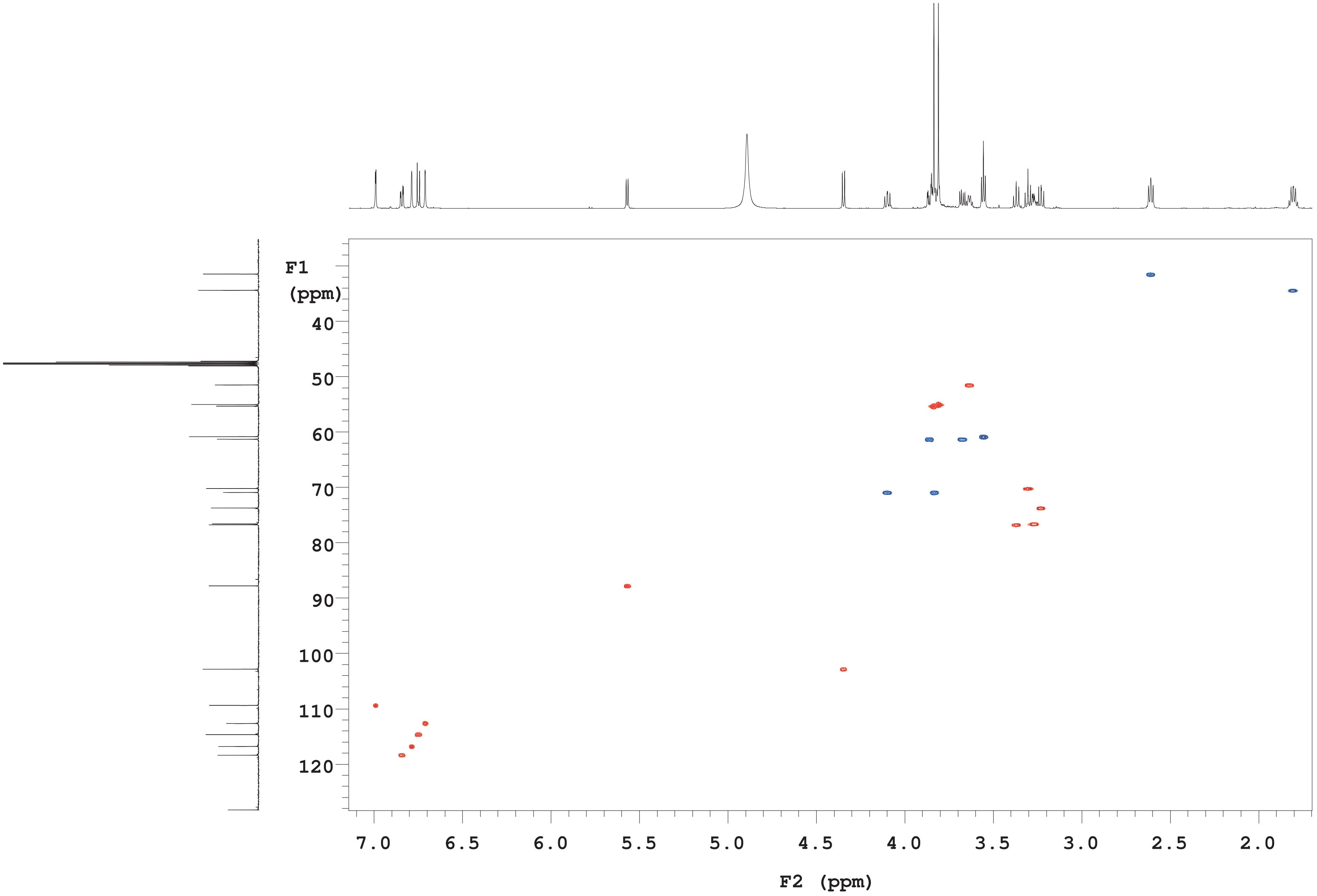
F1
(ppm)
40
50
60
70
80
90
100
110
120
7.0
6.5
6.0
5.5
5.0
4.5
4.0
3.5
3.0
2.5
2.0
F2 (ppm)

Figure. S1C

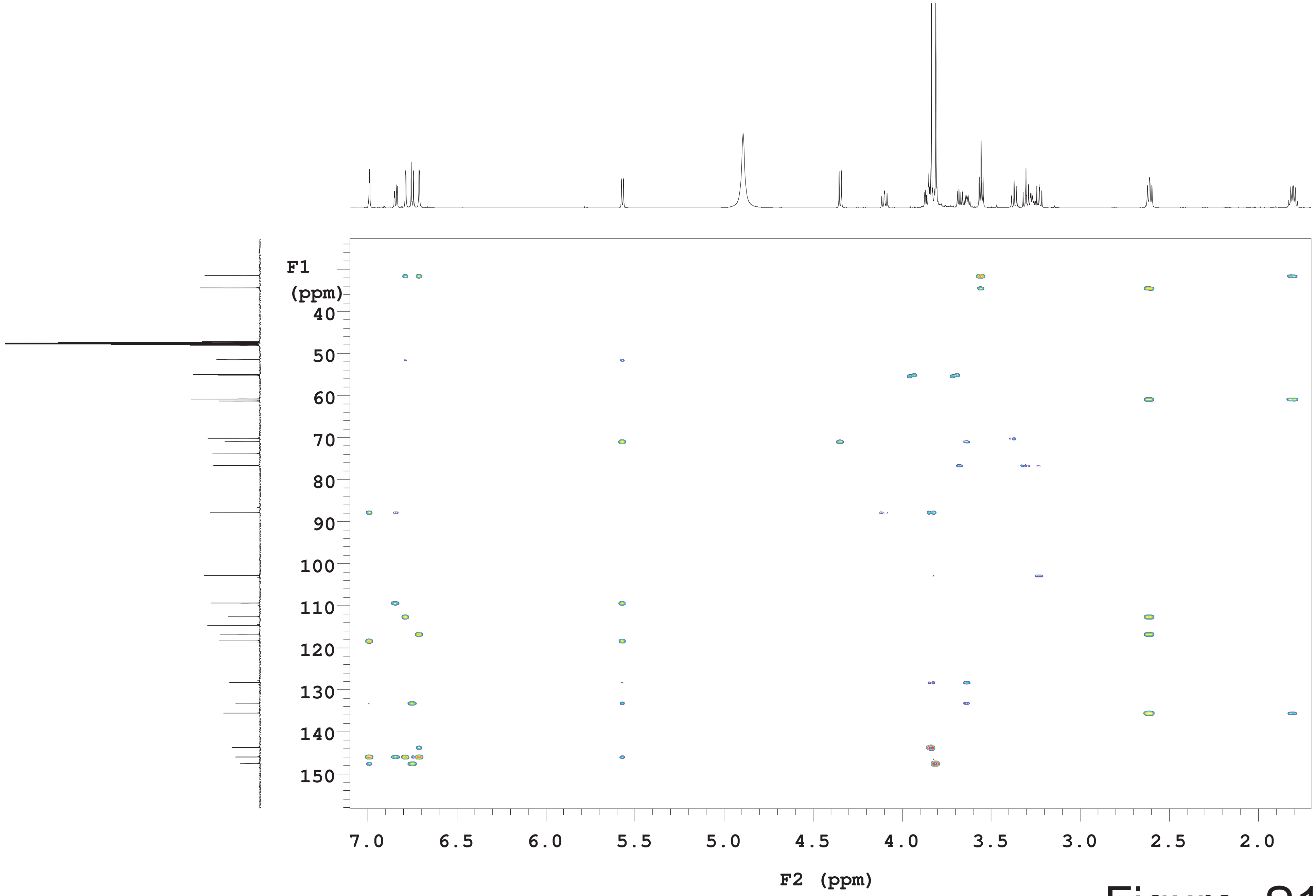
F1 (ppm)
40
50
60
70
80
90
100
110
120
130
140
150
7.0
6.5
6.0
5.5
5.0
4.5
4.0
3.5
3.0
2.5
2.0
F2 (ppm)

Figure. S1D

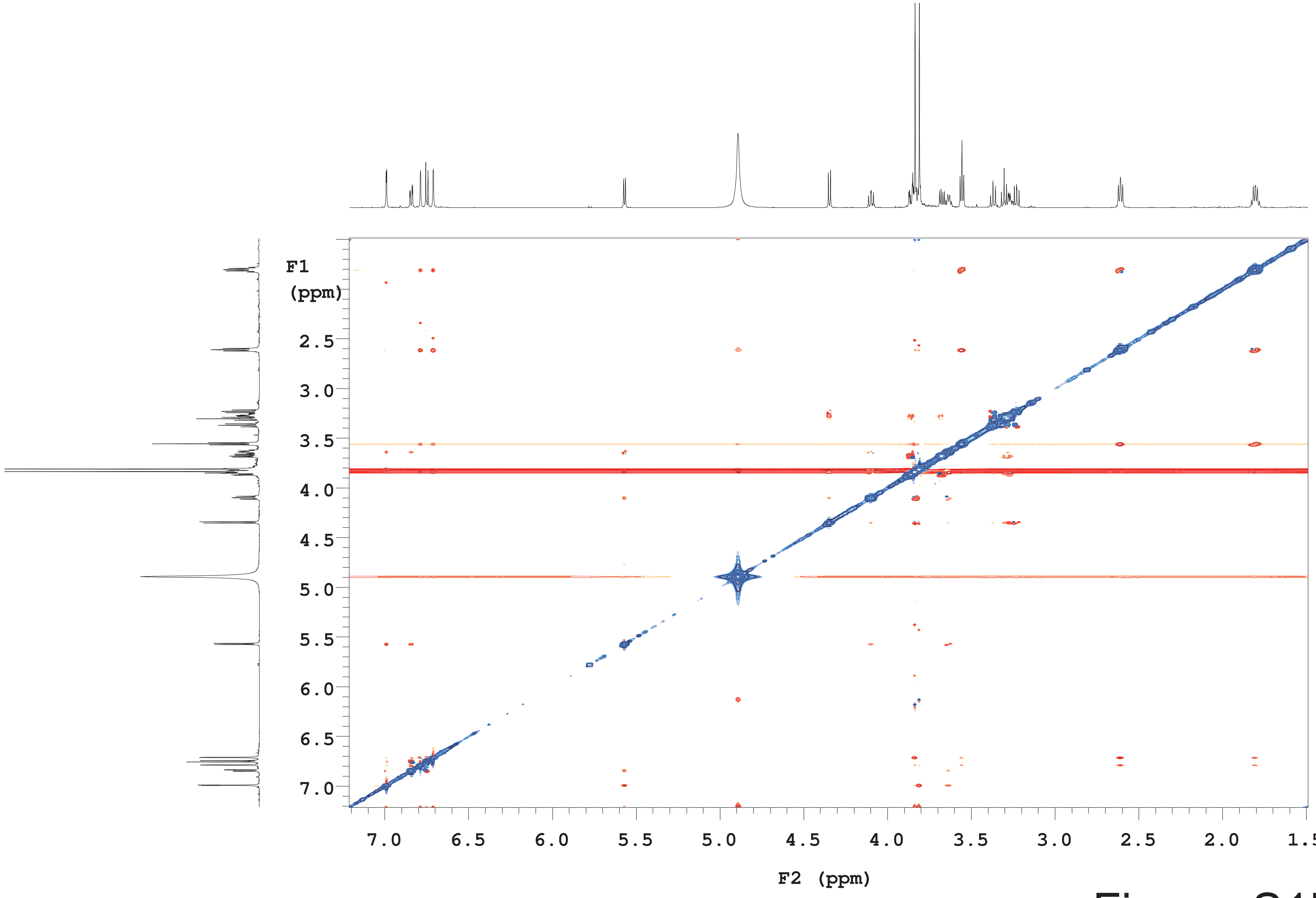
F1
(ppm)
2.5
3.0
3.5
4.0
4.5
5.0
5.5
6.0
6.5
7.0
7.0
6.5
6.0
5.5
5.0
4.5
4.0
3.5
3.0
2.5
2.0
1.5
F2 (ppm)

Figure. S1E

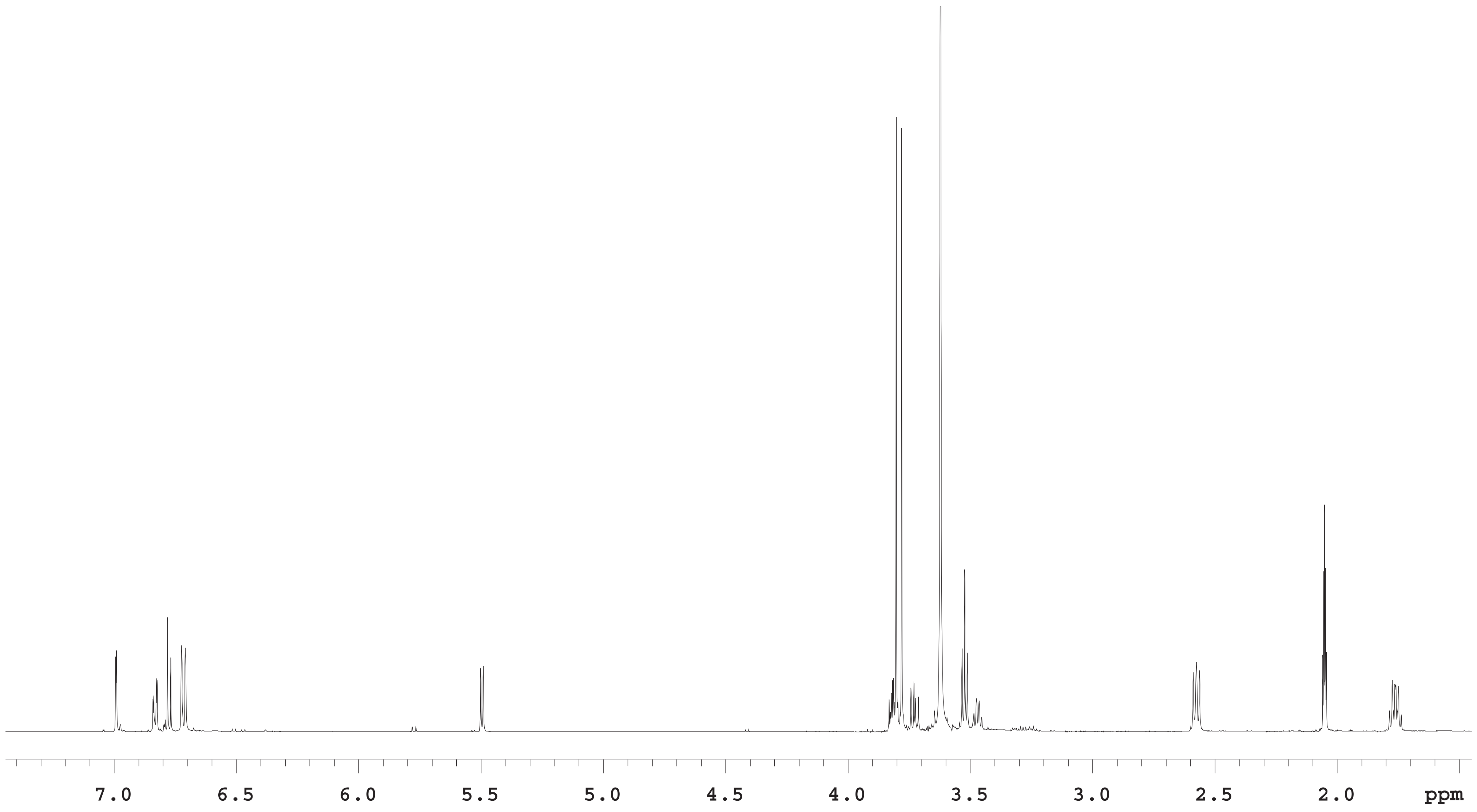

Figure. S2A

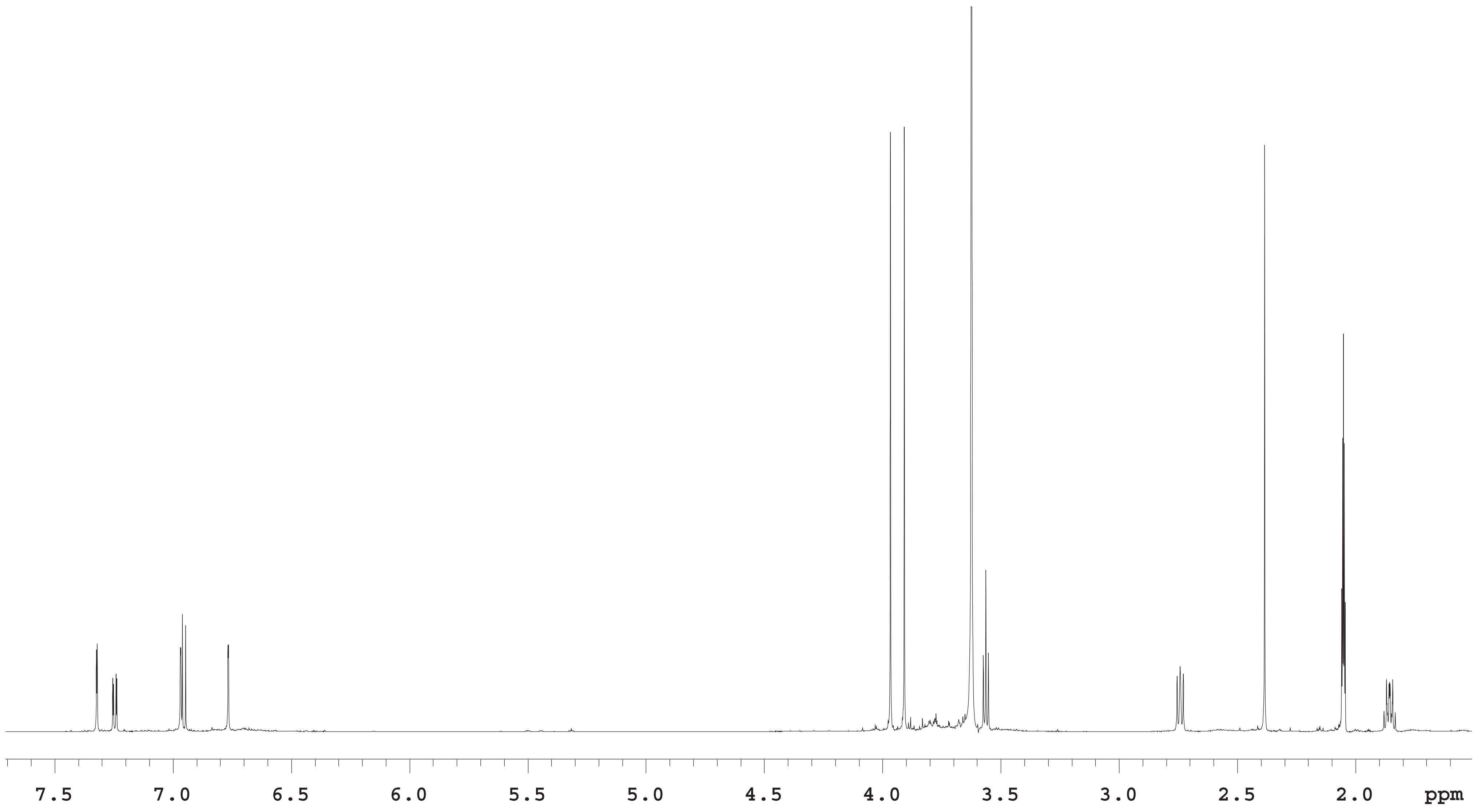

Figure. S2B

Rubuphenol (**11**)

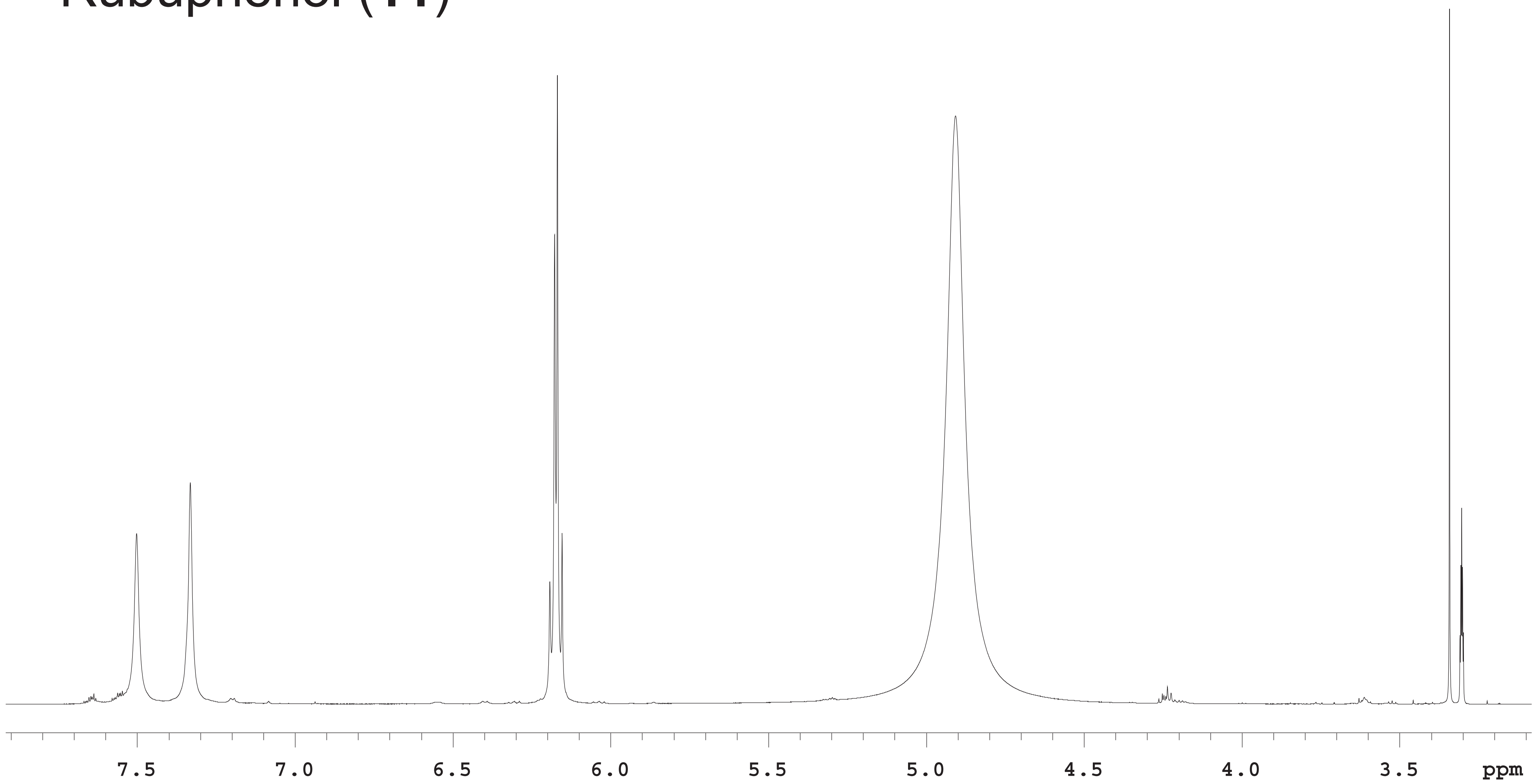

Figure. S3A

Rubuphenol (**11**)

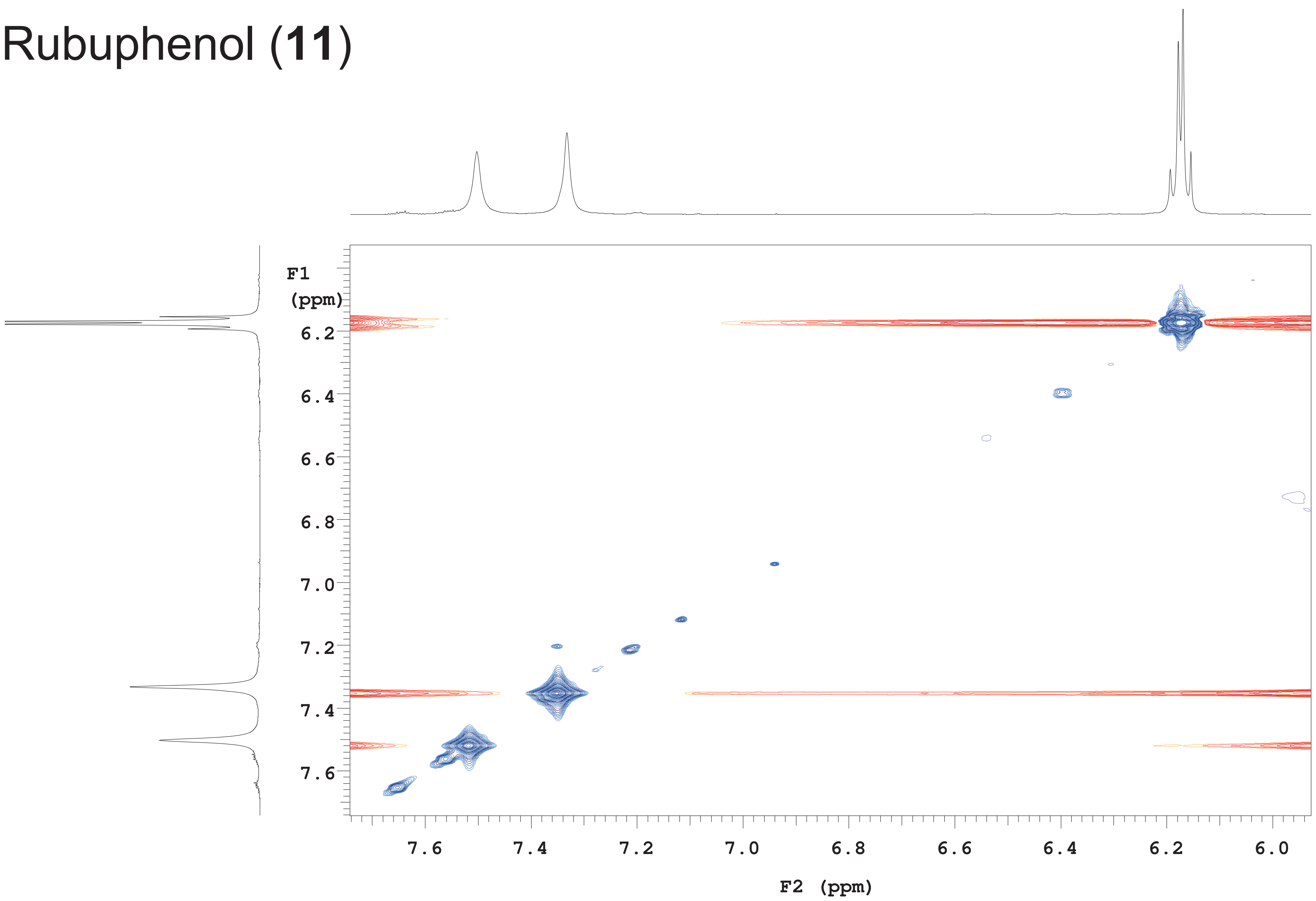

Figure. S3B

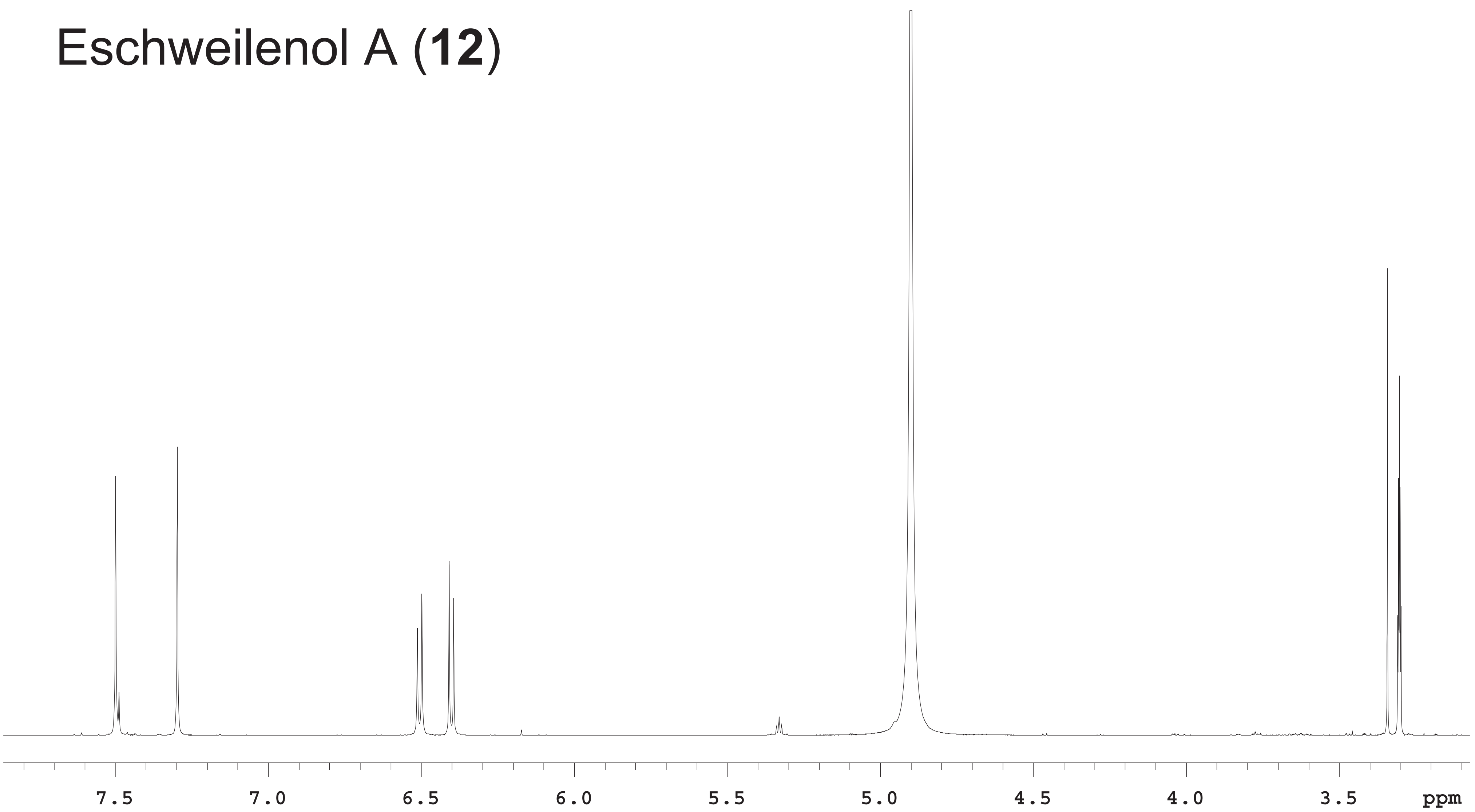

Figure. S3A

Eschweilenol A (**12**)

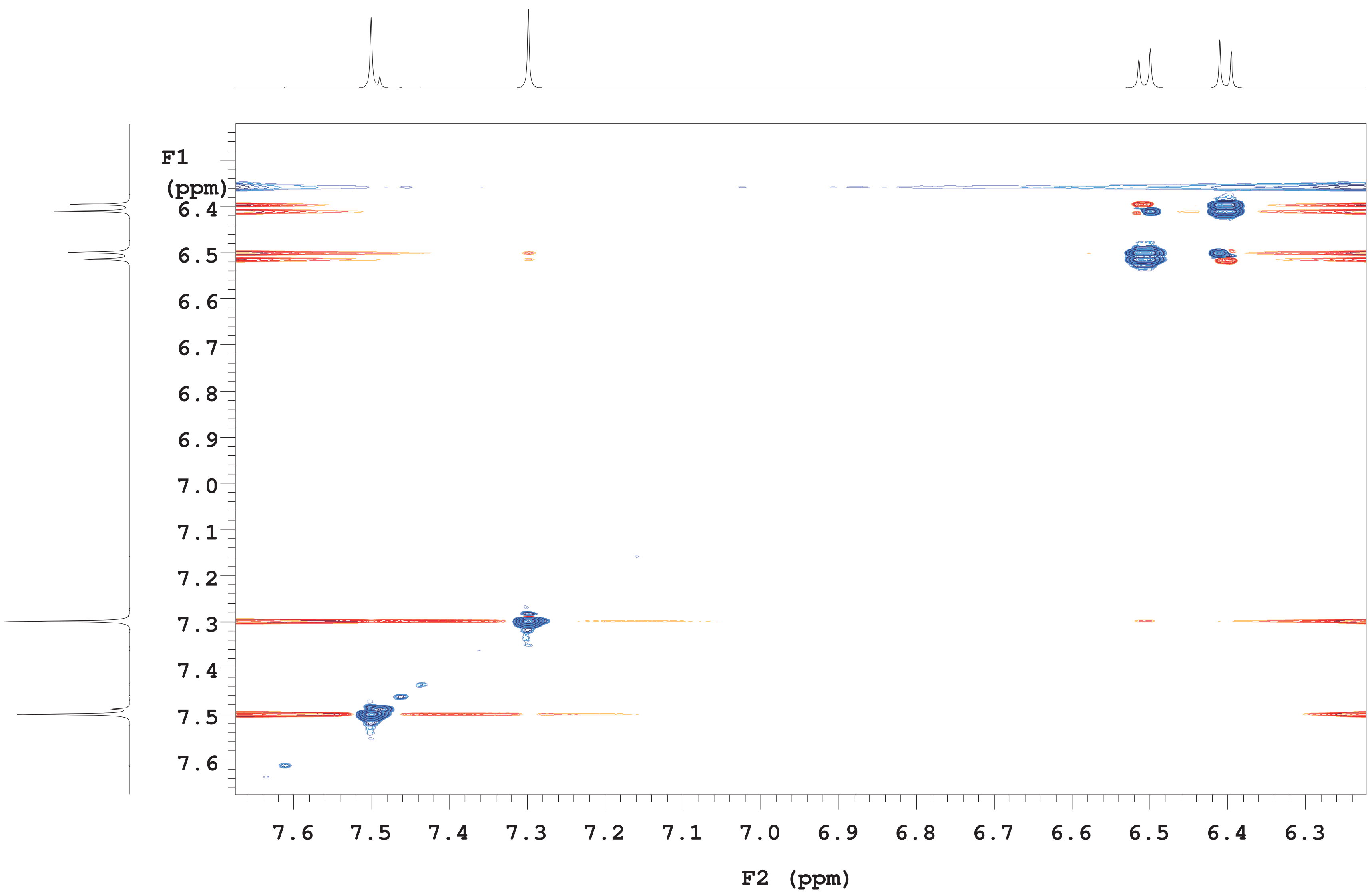

Figure. S3B

Compound **15**

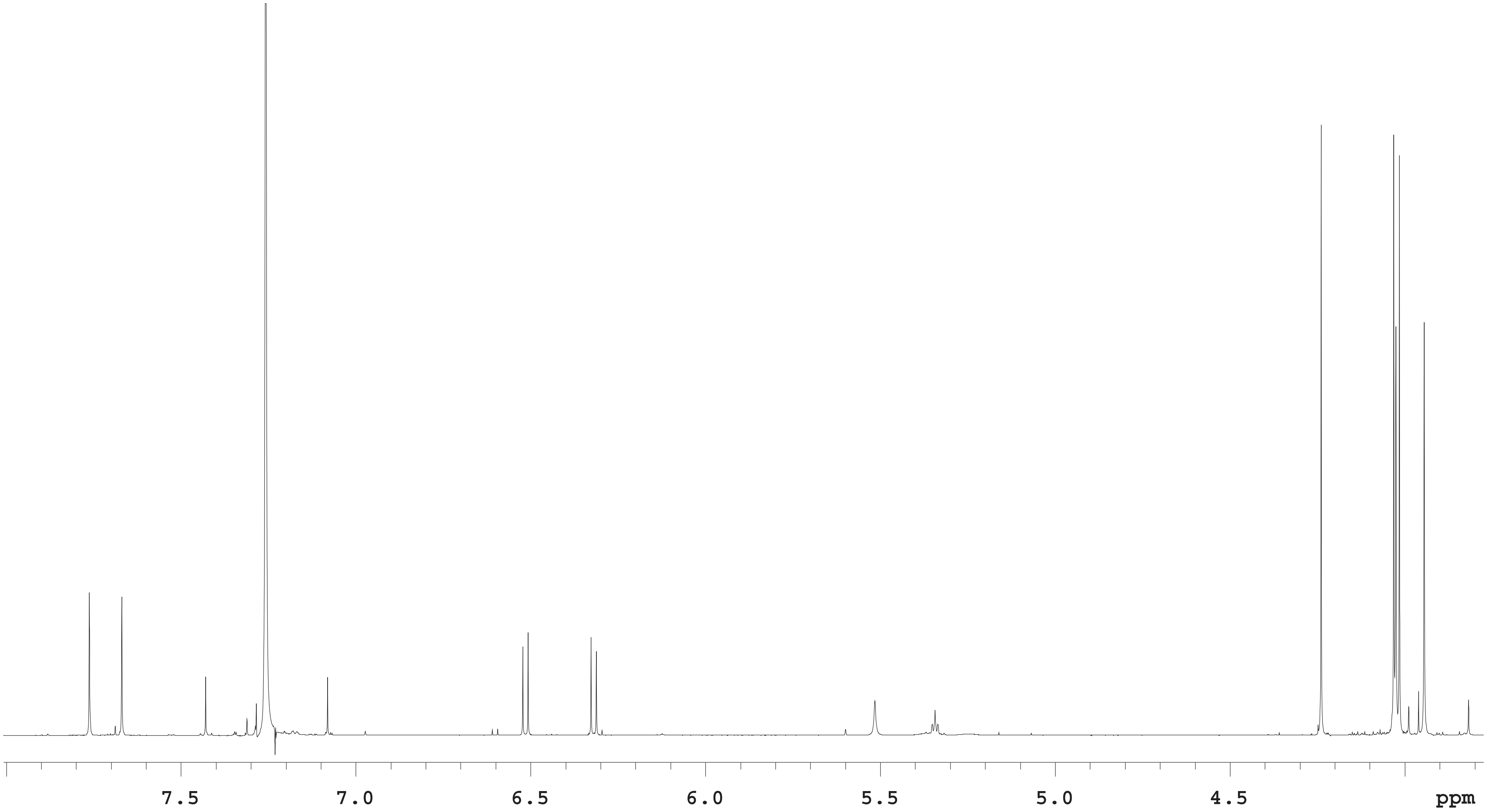

Figure. S4A

Compound **15**

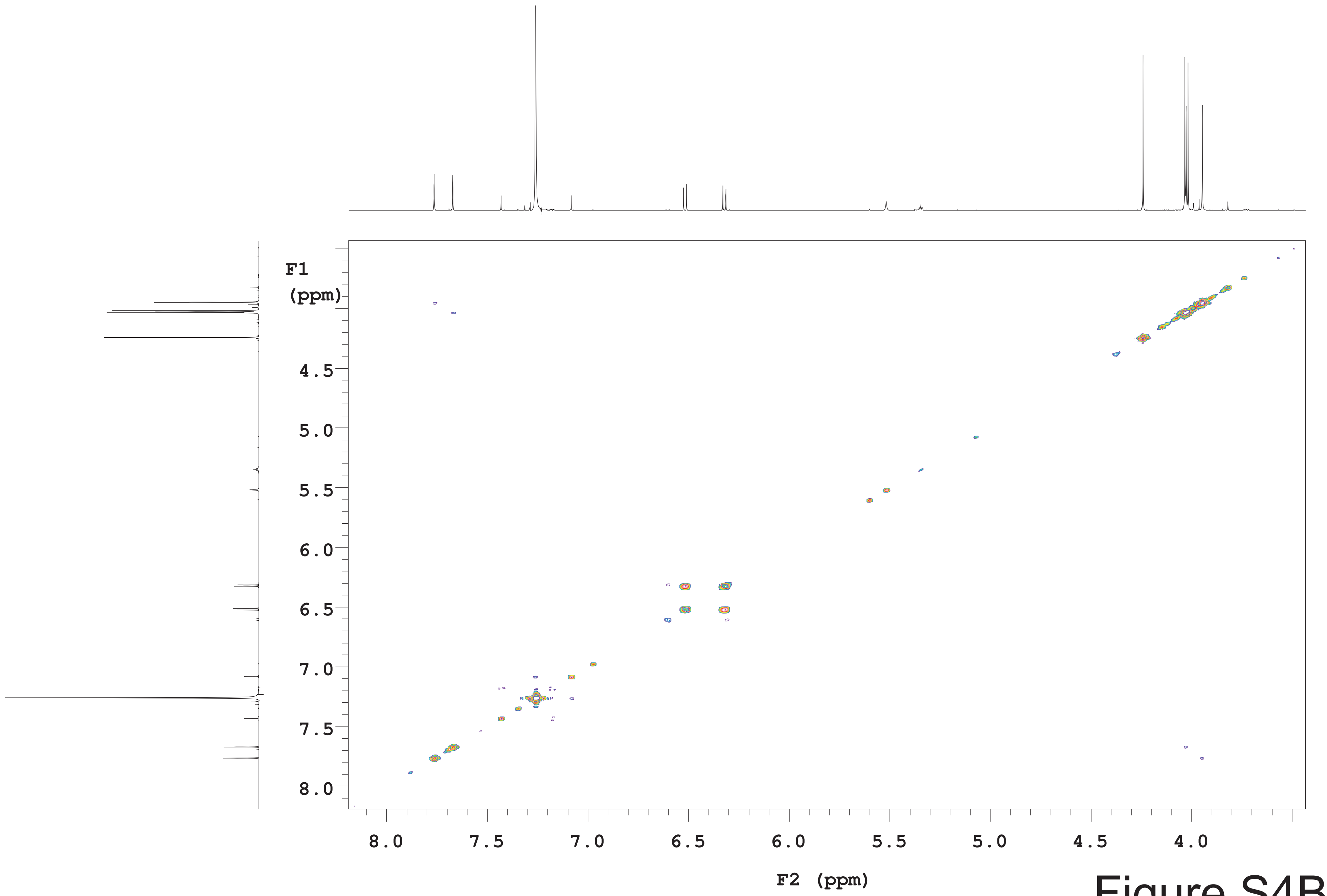

Figure.S4B

Compound **15**

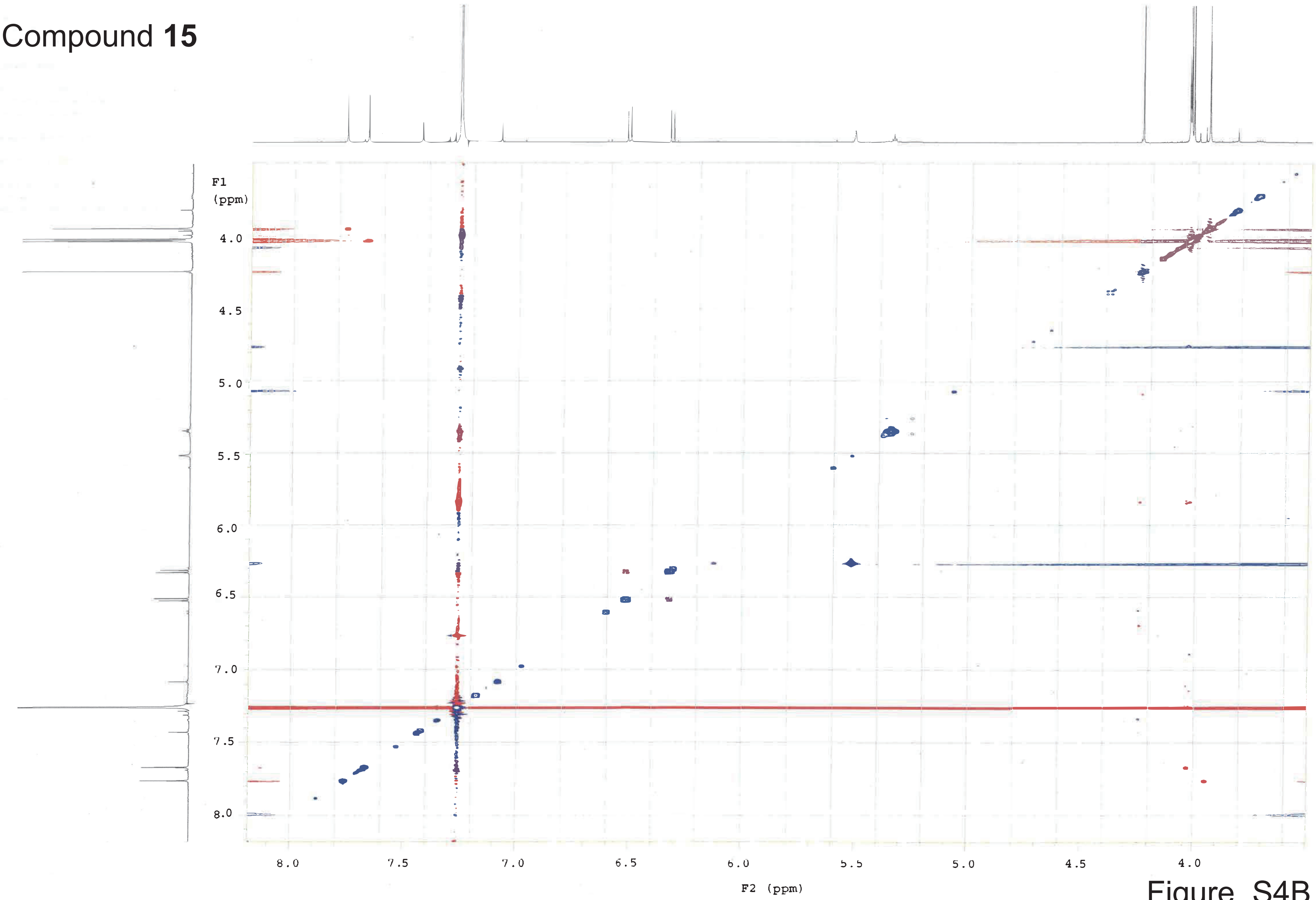

Figure. S4B

Compound **16**

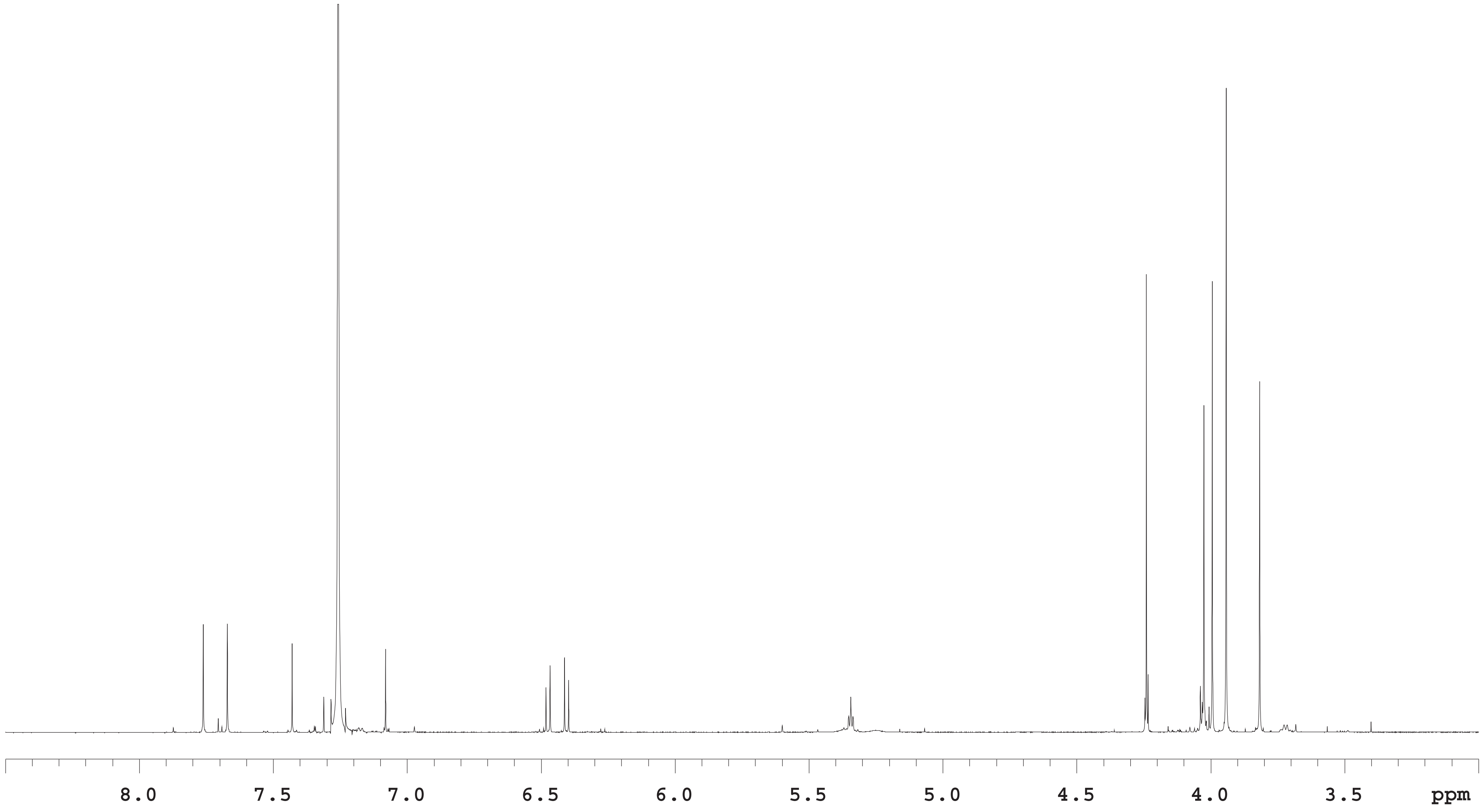

Figure. S4A

Compound **16**

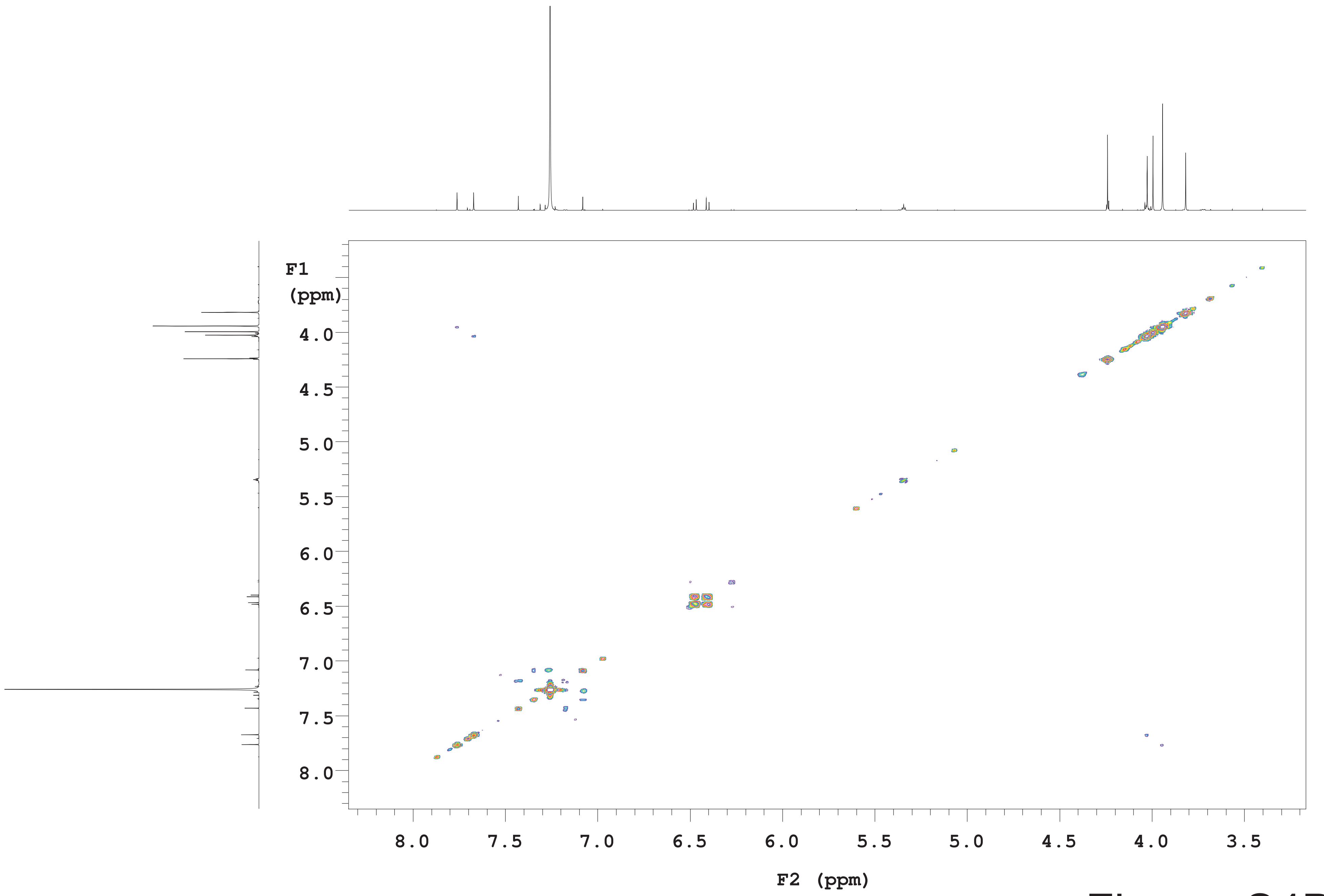

Figure. S4B

Compound **16**

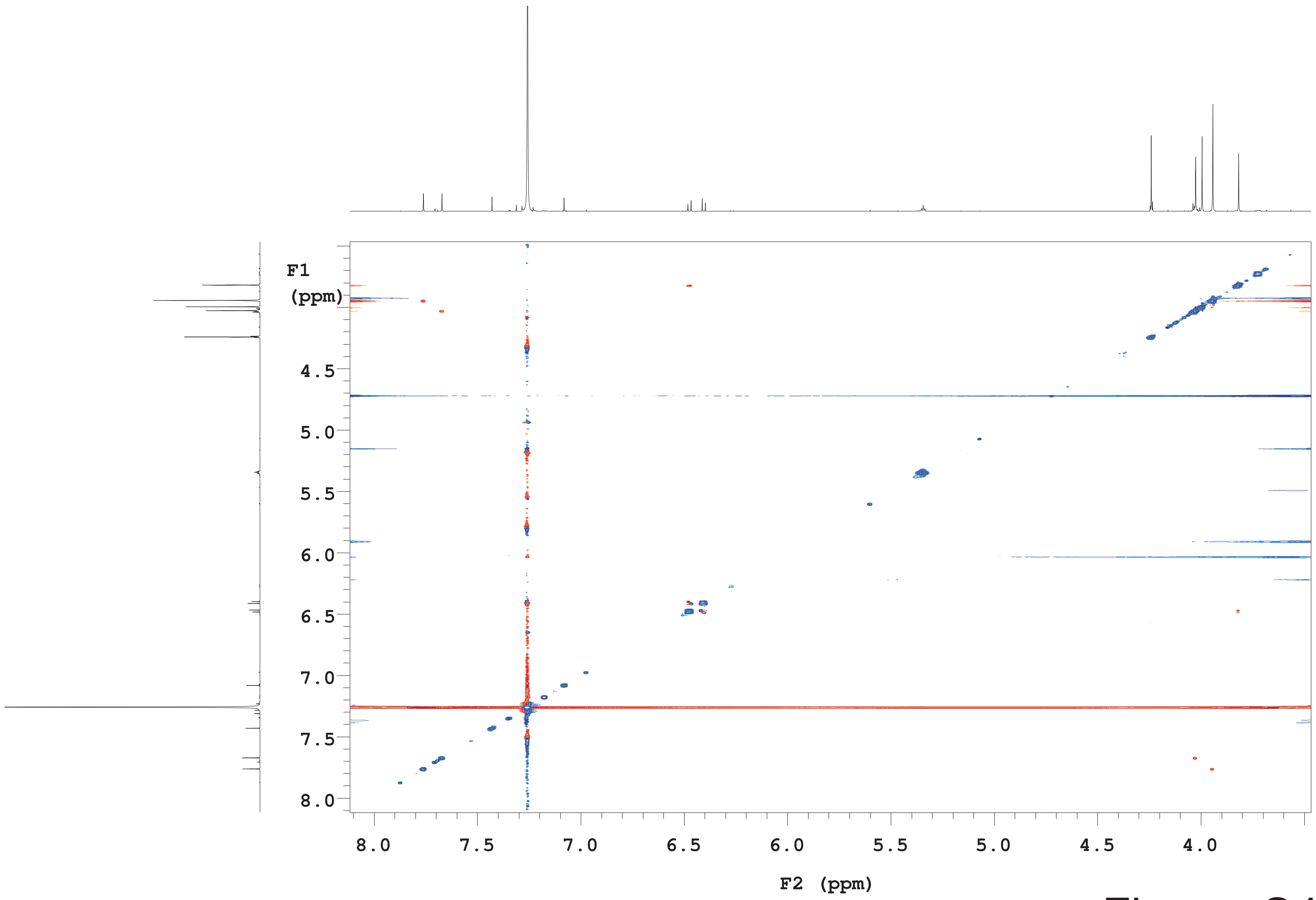

Figure. S4C

Compound **17**

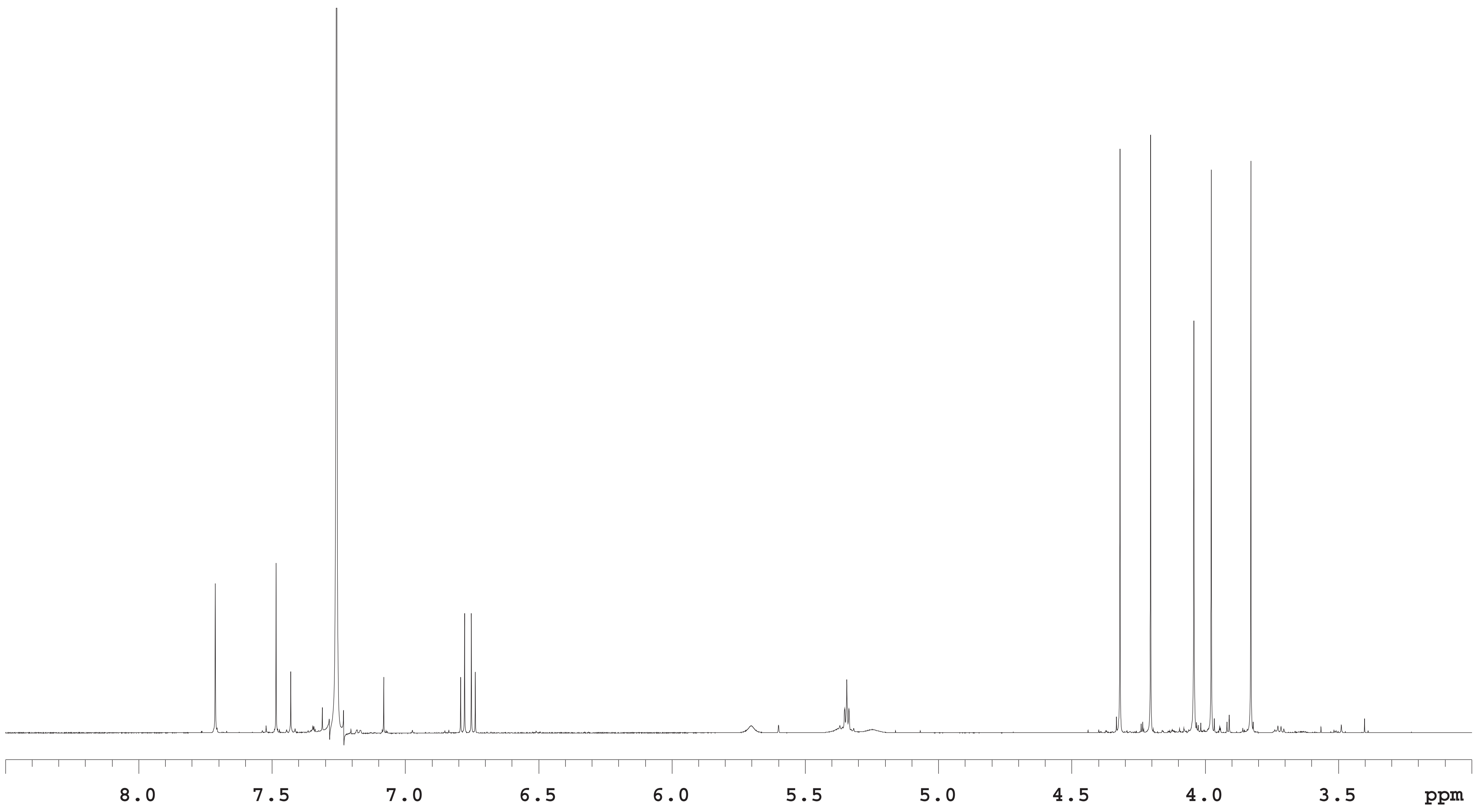

Figure. S4A

Compound **17**

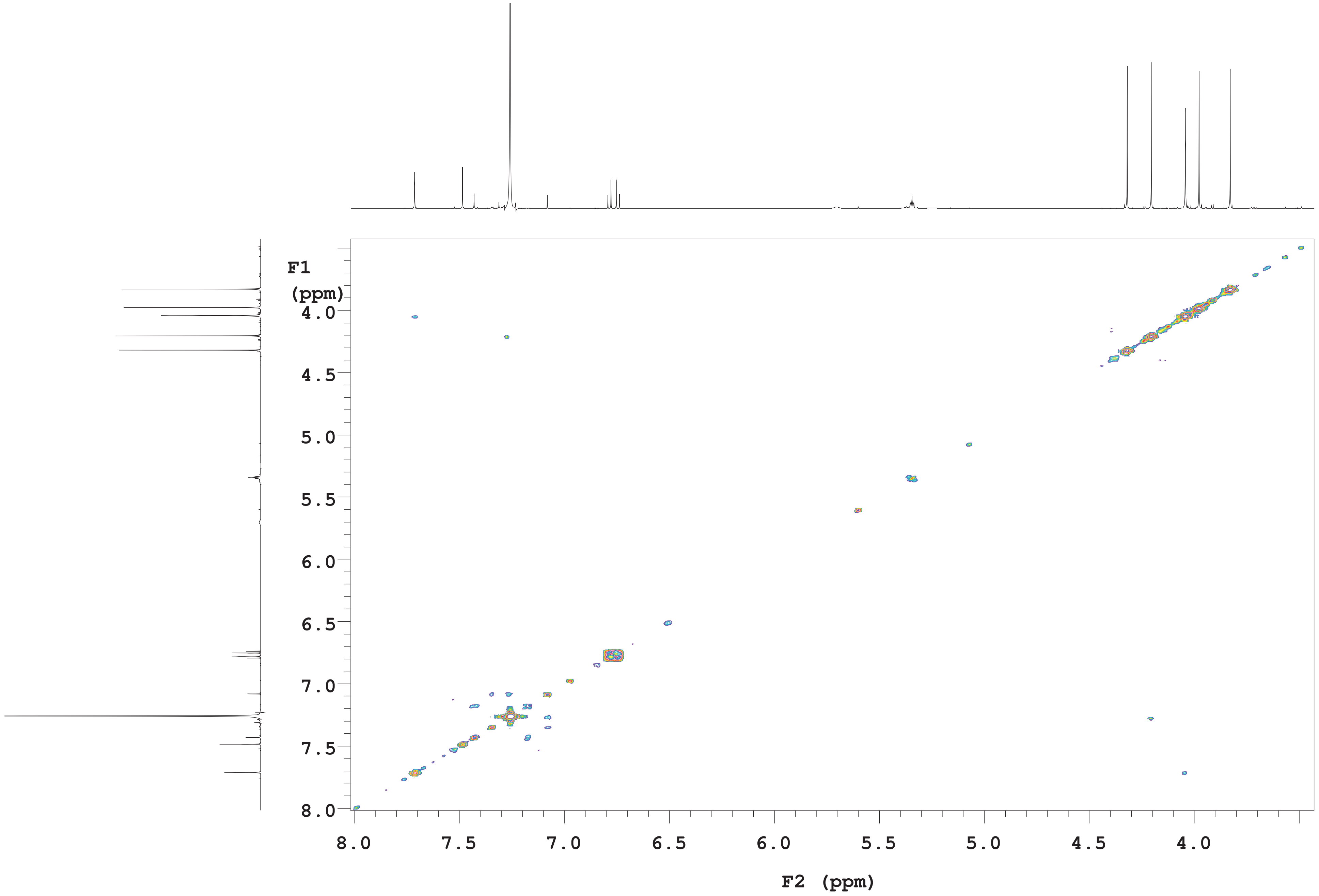

Figure. S4B

Compound **17**

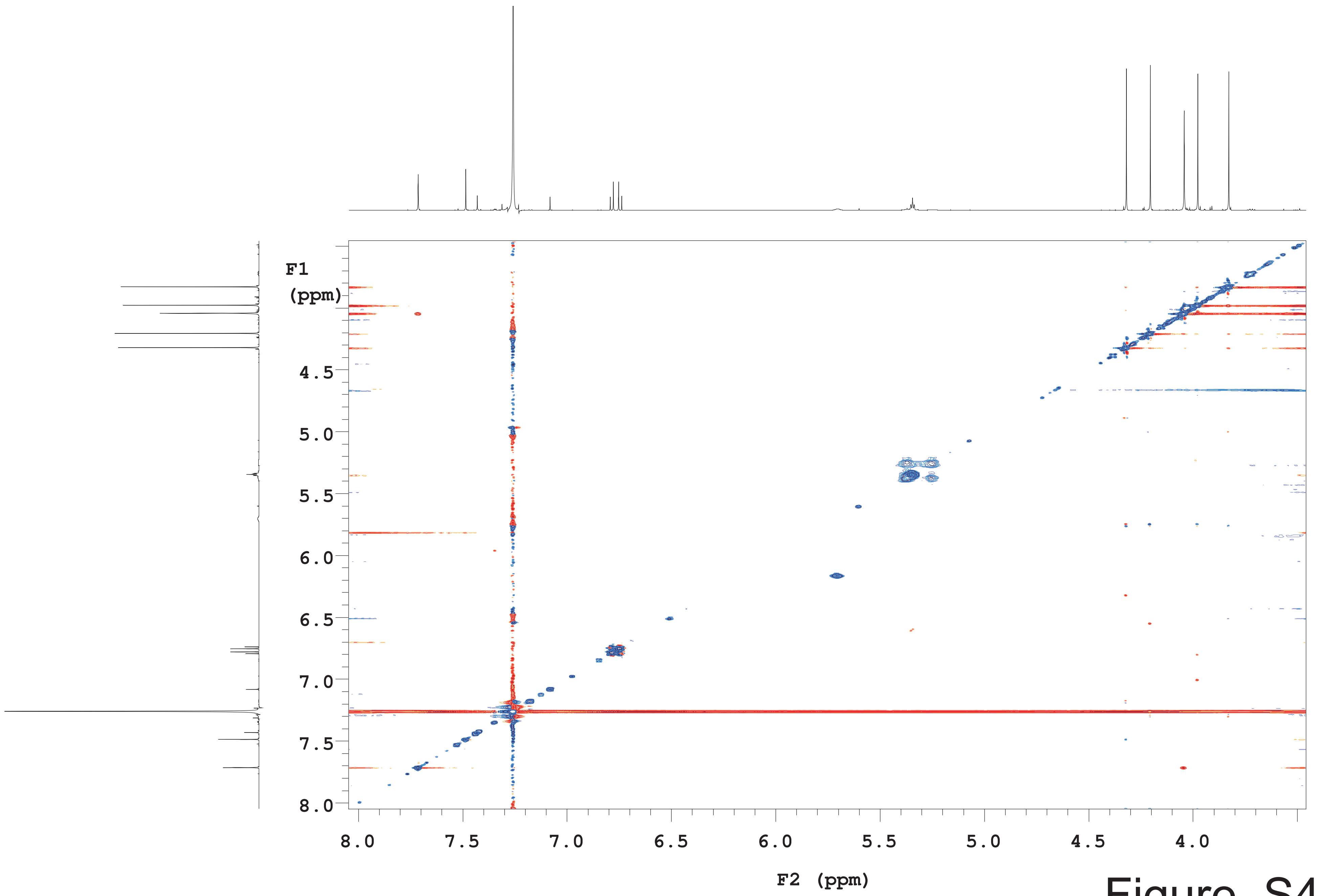

Figure. S4C

Compound **18**

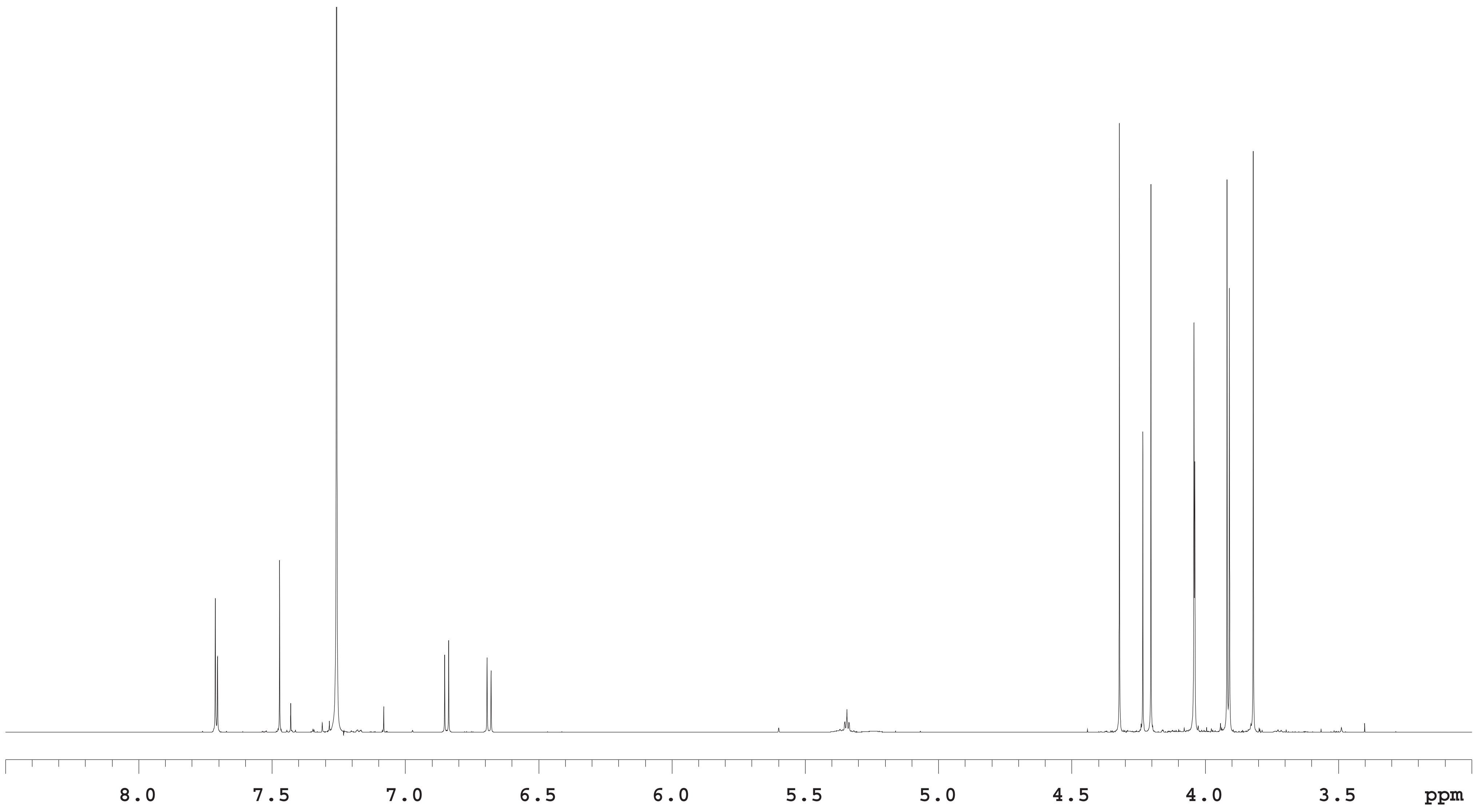

Figure. S4A

Compound **18**

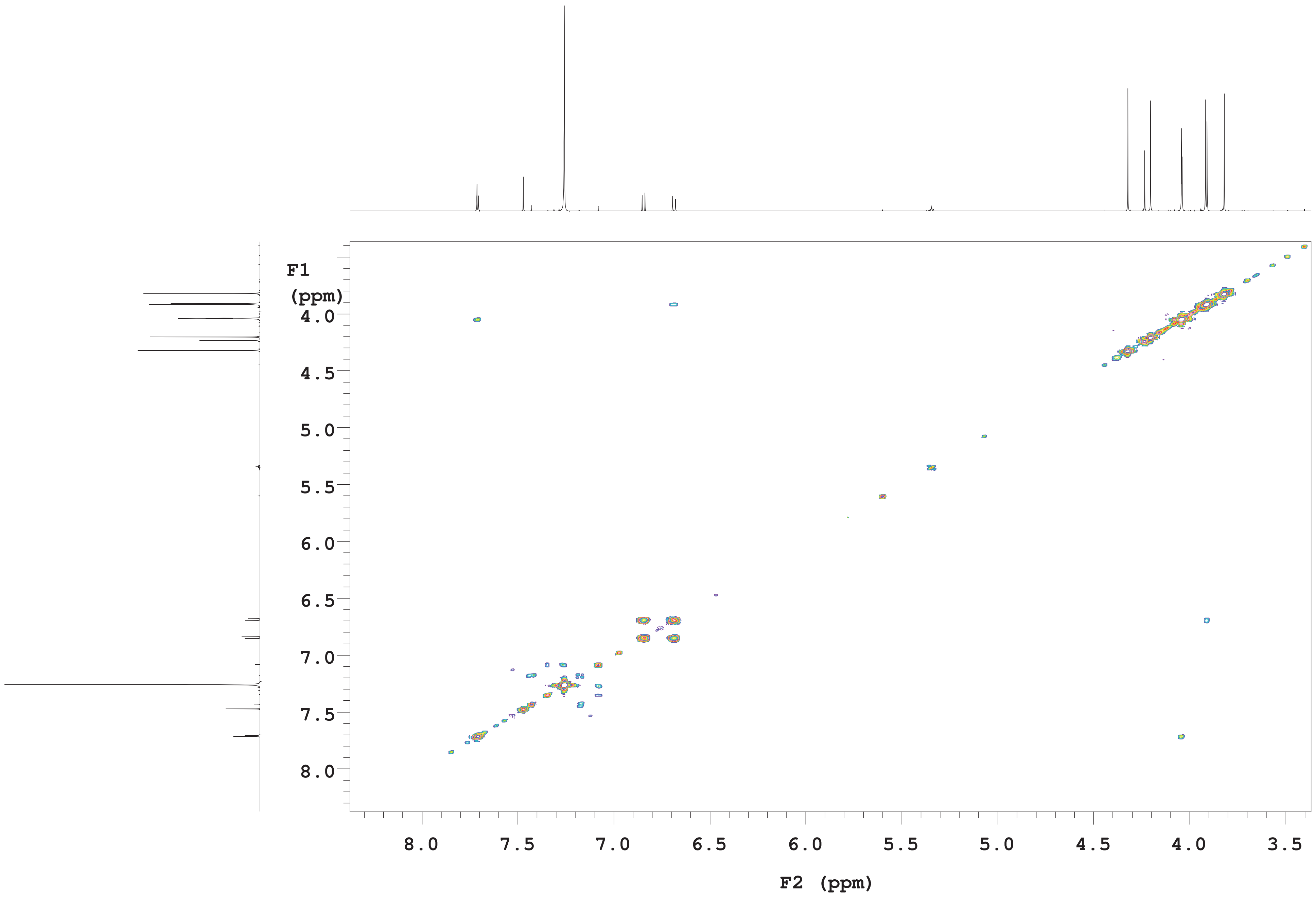

Figure. S4B

Compound **18**

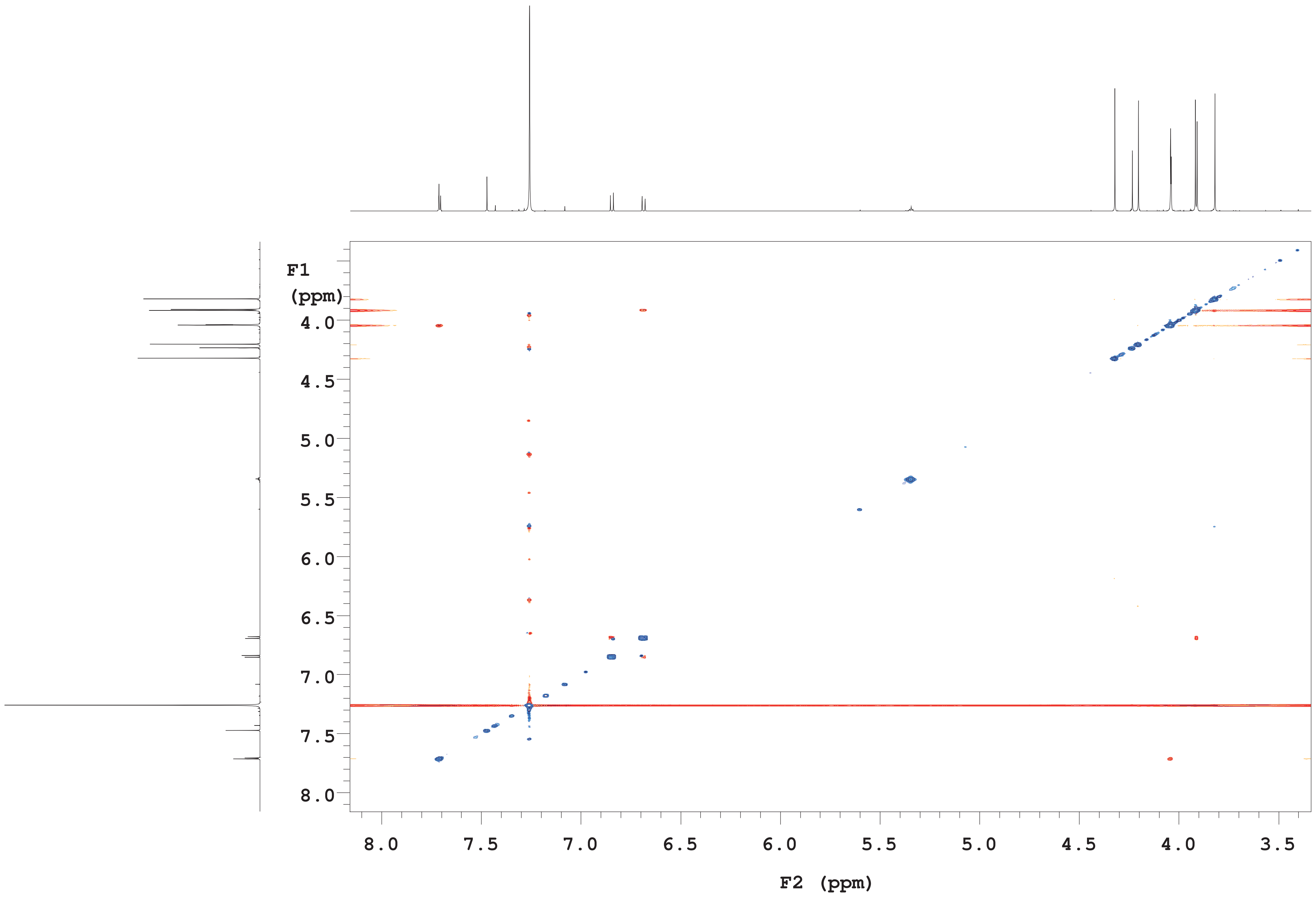

Figure. S4C
